# Alantolactone induces apoptosis, promotes STAT3 glutathionylation and enhances chemosensitivity of A549 lung adenocarcinoma cells to doxorubicin via oxidative stress

**DOI:** 10.1038/s41598-017-06535-y

**Published:** 2017-07-24

**Authors:** Amara Maryam, Tahir Mehmood, He Zhang, Yongming Li, Muhammad Khan, Tonghui Ma

**Affiliations:** College of Basic Medical Sciences, Dalian Medical University, Dalian, Liaoning 116044 People’s Republic of China

## Abstract

Alantolactone (ALT), a sesquiterpene lactone component of *Inula helenium*, has been reported to exert anticancer activity in various cancers. However, the cellular targets and underlying mechanism of anticancer activity of ALT in various cancers including lung cancer has not been fully defined. In the present study, we found that ALT effectively inhibits proliferation and triggers oxidative stress mediated-apoptosis in A549 lung adenocarcinoma cells by inducing ER stress and mitochondrial dysfunction. This ALT-mediated apoptosis was inhibited by NAC while diamide potentiated it. Moreover, ALT effectively suppressed both constitutive and inducible STAT3 activation, inhibited its translocation into nucleus and decreased its DNA binding activity. Further mechanistic study revealed that ALT abrogated STAT3 activation by promoting STAT3 glutathionylation. ROS scavenger NAC reverted ALT-mediated STAT3 glutathionylation and inhibition of STAT3 phosphorylation. Finally, ALT enhanced chemosensitivity of A549 cells to doxorubicin and reversed doxorubicin resistance in A549/DR cells by inhibiting STAT3 activation and P-glycoprotein expression and increasing intracellular accumulation of doxorubicin. Suppression of STAT3 activation by targeting ROS metabolism with ALT thus discloses a previously unrecognized mechanism underlying the biological activity of ALT. Taken together; ALT induces oxidative stress-dependent apoptosis, inhibits STAT3 activation and augments doxorubicin toxicity in A549 lung cancer cells. These findings provide an in-depth insight into the molecular mechanism of ALT in the treatment of lung cancer.

## Introduction

Lung cancer is the most common malignancy and leading cause of cancer-related deaths both in men and women worldwide with approximately 1.4 million deaths annually^1^, out of which 0.6 million deaths occur only in China^2^. Non-small cell lung cancer (NSCLC) is the most common cancer which represents about 80% of all lung cancer cases^1^. At present, the available treatment options for NSCLC include surgery, radiotherapy, chemotherapy, targeted therapy and/or a combination of these therapies depending upon the stage of cancer^3^. Surgery remains the best treatment option for early stage (stage I and II) NSCLC. Unfortunately, more than 70% cases of NSCLC are diagnosed at an advanced stage^3^. Thus, only about 25–30% cases of NSCLC are suitable for an effective surgical resection^4^. Radiotherapy, chemotherapy and targeted therapy are recommended treatment options for advanced stage NSCLC^3^. Despite concerted efforts to improve the current therapies, the prognosis of NSCLC remains very poor with 5 years survival rate about 15%^1^. Epidermal growth factor receptor (EGFR) has been frequently found mutated and over-expressed in NSCLC^5, 6^. In the last two decades, several EGFR-directed tyrosine kinase inhibitors (TKIs) have been developed. Some of these TKIs such as gefitinib, erlotinib and afatinib have been approved by FDA for the treatment of NSCLC^6, 7^. Despite initial efficacy, development of secondary drug resistance has become the major limitation of these drugs^7, 8^. Doxorubicin, a highly potent clinical anticancer drug (Topoisomerase II inhibitor) is widely used to treat various human malignancies including breast cancer, bladder cancer, multiple myeloma and small cell lung cancer (SCLC)^9^. Unlike its high activity against SCLC, it remains ineffective against NSCLC^9^. Therefore, it is important to explore novel strategies to enhance the activity of doxorubicin against NSCLC.

Recent research has shown that prevailing genotype-based targeted therapies for NSCLC induce signal transducer and activator of transcription 3 (STAT3) activation^6, 10, 11^. Accumulating evidence from multitudinous studies has shown that aberrant expression and activation of STAT3 is implicated in tumor progression, drug resistance and metastasis of various human cancers including NSCLC^6, 12, 13^, suggesting that STAT3 may contribute to resistance to prevailing chemotherapy in NSCLC. STAT3 is activated by phosphorylation at tyrosine 705 (Y705) or serine 727 (S727) and its activation is regulated by multiple pathways including cytokine receptors, receptor tyrosine kinases (RTKs), non-receptor tyrosine kinases (nRTKs), G-protein coupled receptors (GPCRs), Toll like receptors and protein phosphatases^14^. There are now sound evidences that inhibition of one RTK allows cancer cells to survive using another RTK^8^. As STAT3 is one of the major RTKs’ downstream transcription factors, exploring novel chemotherapeutic agents able to effectively inhibit constitutive as well as inducible activation of STAT3 from multiple stimuli through biochemical modification hold greater potential to reduce cancer mortality.

Given the acquired drug resistance, genomic instability and aberrant activation of signaling pathways associated with targeted anticancer drugs developed in the past by exploiting the genetic differences between cancer cells and normal cells, it is necessary now to explore alternative novel anticancer strategies that can effectively kill cancer cells. It is well established now that cancer cells contain higher level of reactive oxygen species (ROS) as compared to normal cells which plays important role in cancer cell survival and proliferation. Recent research has shown that this unique biochemical property of cancer cells can be exploited for therapeutic benefits^15–17^.

ROS act as double-edged sword in living cells. At low level, ROS act as signaling molecules and play a vital role in various biological processes including cell survival, proliferation, differentiation and gene expression, at higher level however; ROS exert oxidative stress and induce cell death through various signaling pathways^15^. Killing cancer cells by exploiting this biochemical vulnerability of cancer cells with ROS-targeting phytochemicals has been shown to be feasible in various *in vitro* and *in vivo* experimental models^16, 18–20^. ROS-targeted anticancer drug development strategy holds the promise to set the cancer on the road to ruin as it can be applied more broadly against various human cancers of multiple origins irrespective of their genotype and are less likely to suffer from drug resistance. Therefore, it is dire need to explore the exact molecular mechanism of ROS based treatment for the future development of highly effective anticancer drugs.

Alantolactone (ALT), a major bioactive sesquiterpene lactone component of *Inula helenium* has been reported to possess multiple biological and pharmacological activities including antibacterial, antifungal, anti-inflammatory and anticancer effects^21^. In recent years, ALT has attracted the attention of researchers due to its potential anticancer activity against various human cancer cells through multiple mechanisms^21–24^. We have previously shown that ALT induces ROS-mediated apoptosis in U87 glioblastoma and HepG2 liver cancer cells^21, 25^. Moreover, we and Chun *et al*., have recently shown that ALT inhibits STAT3 activation in liver^25^ and breast cancer^23^ cells, respectively. However, the exact molecular mechanism by which ALT inhibits STAT3 activation remains largely unexplored. In the present study, we found that ALT primarily induces oxidative stress resulting in ER stress, mitochondrial dysfunction, and inhibition of STAT3 activation which ultimately lead to apoptotic cell death in A549 lung cancer cells. Inhibition of STAT3 activation by ALT through S-glutathionylation has revealed an unprecedented mechanism and provided in-depth insight into molecular mechanism of ALT in the treatment of A549 lung adenocarcinoma cells as a single agent or in combination with clinical drugs suffering from drug resistance due to induction of STAT3 activation.

## Results

### ALT inhibits proliferation and induces cell death in lung adenocarcinoma cells

**Figure 1:**
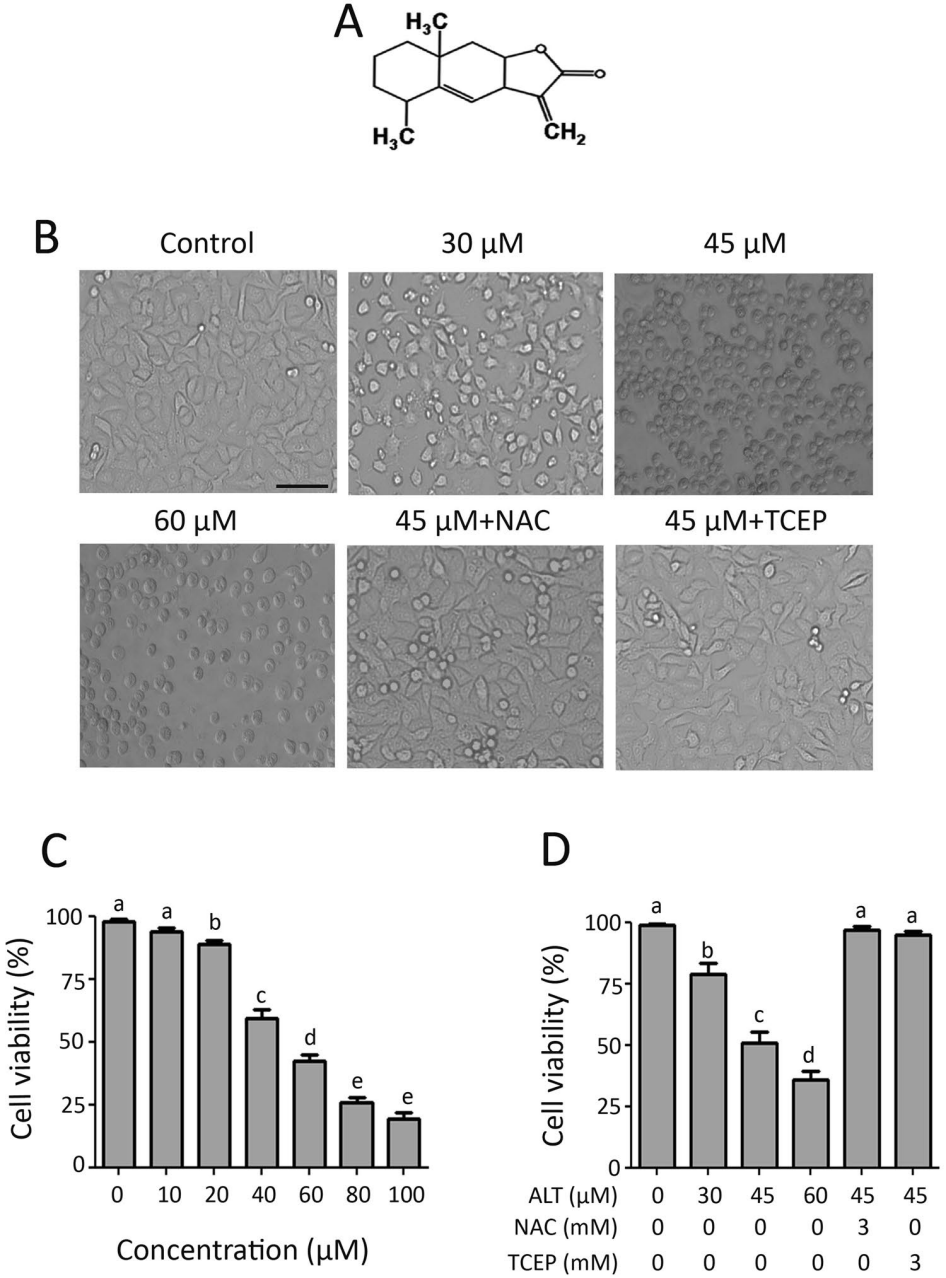
Effect of ALT on morphology and viability of cells. (**A**) Chemical structure of ALT. (**B**) A549 cells were treated with indicated concentrations of ALT for 12 h and cells morphological changes were observed under phase-contrast microscope. Scale bar = 100 μm. Pretreatment of cells with 3 mM NAC and/or 3 mM TCEP completely suppressed the toxic effect of ALT. (**C**) Cells were treated with 10–100 μM ALT for 12 h and cell viability was measured by MTT assay. Data are expressed as Mean ± SEM (n = 3). Columns not sharing same superscript letters differ significantly (*P* < *0*.*05*). (**D**) A549 cells were treated with indicated concentrations of ALT for 12 h and live and dead cells were quantified using Live/dead assay. Pretreatment of cells with NAC and/or TCEP reversed ALT-induced death in A549 cells. Data are expressed as Mean ± SEM (n = 3). Columns not sharing same superscript letters differ significantly (*P* < *0*.*05*).

The cytotoxicity of ALT against NSCLC was evaluated using A549 and NCI-H1650 cells. ALT decreased viability of A549 cells in a dose-dependent manner as evident from Fig. 1C. The IC_50_ value of ALT against A549 cells was found to be 45 μM at 12 h time point. As shown in Fig. 1B, ALT induces severe morphological changes characteristically associated with cell death such as rounding up, presence of cell debris in culture media and reduction in total number of cells in a dose-dependent manner. To further confirm ALT-induced cell death, we performed live/dead assay by staining the cells with calcein AM and PI. Being cell membrane permeable, live cells take up calcein AM, de-esterify and retaine the calcein dye inside while dead cells cannot retain calcein and are stained only with PI. As shown in Fig. 1D, ALT induced cell death in A549 cells in a dose-dependent manner. Pretreatment of cells with 3 mM NAC and/or TCEP completely suppressed the toxic effects of ALT indicating involvement of oxidative stress in ALT-induced toxicity in A549 cells (Fig. 1B and D). A similar oxidative stress mediated cytotoxic effect of ALT was observed in NCI-H1650 lung cancer cells (Supplementary Figure 1A).

**Figure 2:**
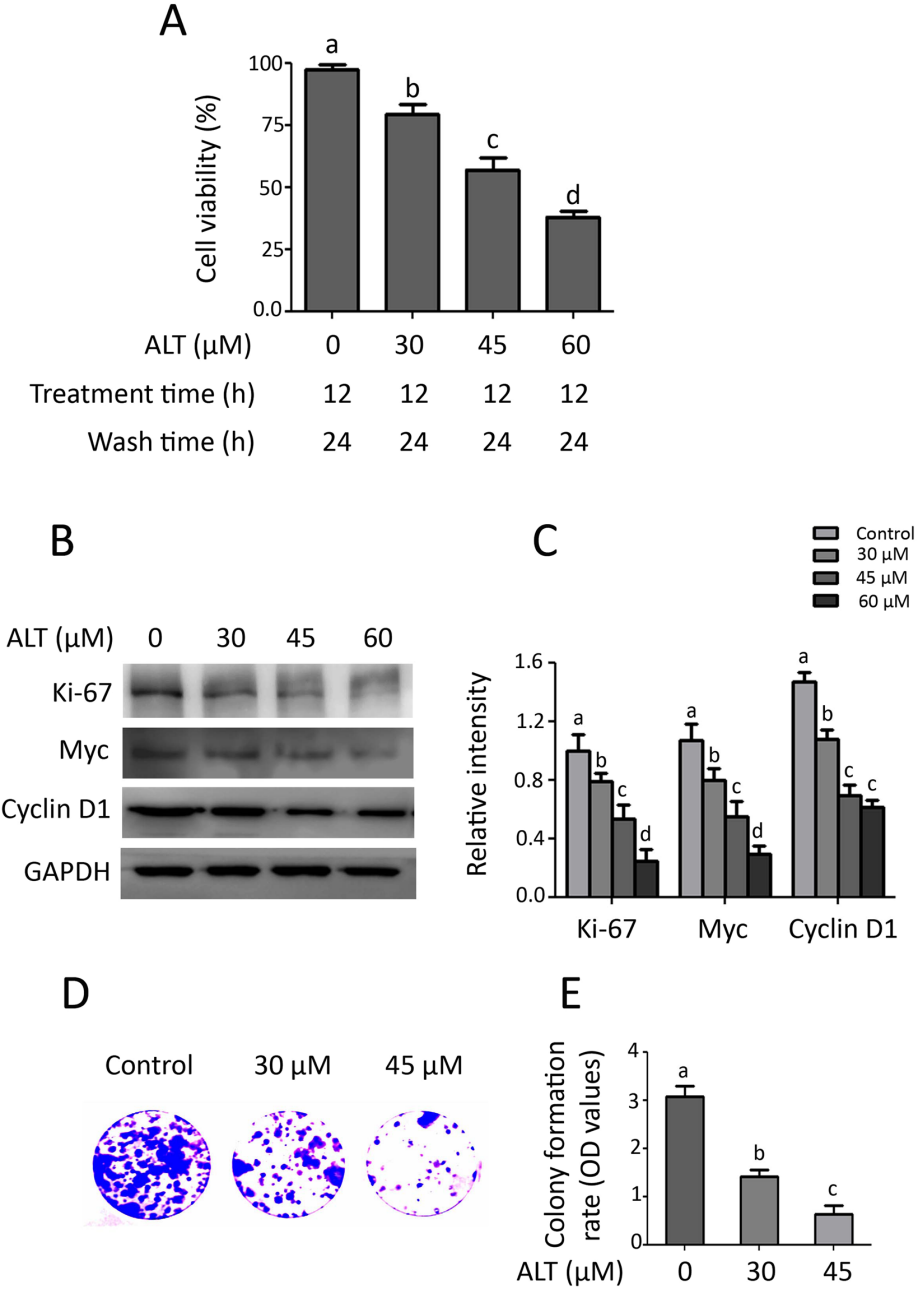
Effect of ALT on cell proliferation. (**A**) A549 cells were seeded in 96 well plates and incubated at 37 °C overnight. The cells were treated with indicated concentration of ALT for 12 h. Following treatment, drug containing medium was replaced with fresh medium and cells were allowed to grow in drug free medium for 24 h. The cell proliferation was measured by MTT assay. (**B**) A549 cells were treated with ALT for 12 h and expression of Ki-67, myc, cyclin D1 was measured by Western blot. (**C**) Statistical analysis of data from B. Columns not sharing same superscript letters within the same group differ significantly (*P* < *0*.*05*). (**D**) Cells treated with indicated concentration of ALT for 12 h were seeded into 6 well plates and allowed to grow into colonies for 7 days. The colonies were fixed, stained with crystal violet, washed and photographed. (**E**) To quantify proliferation rate, methanol was added to each well to dissolve crystal violet stain and absorbance was measured at 595 nm. Columns not sharing same superscript letters within the group differ significantly (*P* < *0*.*05*).

To further evaluate the effect of ALT on cell proliferation, we treated the cells with indicated concentrations of ALT for 12 h in 96 well plates. Following treatment, drug containing medium was replaced with fresh medium and cells were allowed to grow in drug free medium at 37 °C for another 24 h. Finally, the proliferation of cells was measured by MTT assay. The data demonstrated that ALT inhibited proliferation of cells in a dose-dependent manner (Fig. 2A). To further probe into underlying anti-proliferative mechanism, we measured the expressions of various proliferation markers such as Ki-67, Myc and cyclin D1 by Western blot. The data demonstrated that ALT effectively decreased the expressions of Ki-67, Myc and cyclin D1 in a dose-dependent fashion (Fig. 2B and C). Growth inhibitory effect of ALT on A549 cell proliferation was also evaluated by colony formation assay using relatively less toxic concentrations (30 & 45 μM). Consistent with results from MTT assay, ALT remarkably suppressed colony formation in a dose-dependent manner (Fig. 2D and E). Collective data demonstrate that ALT exerts irreversible growth inhibitory effect in A549 lung cancer cells.

### ALT induces oxidative stress-mediated apoptosis in lung adenocarcinoma cells

**Figure 3:**
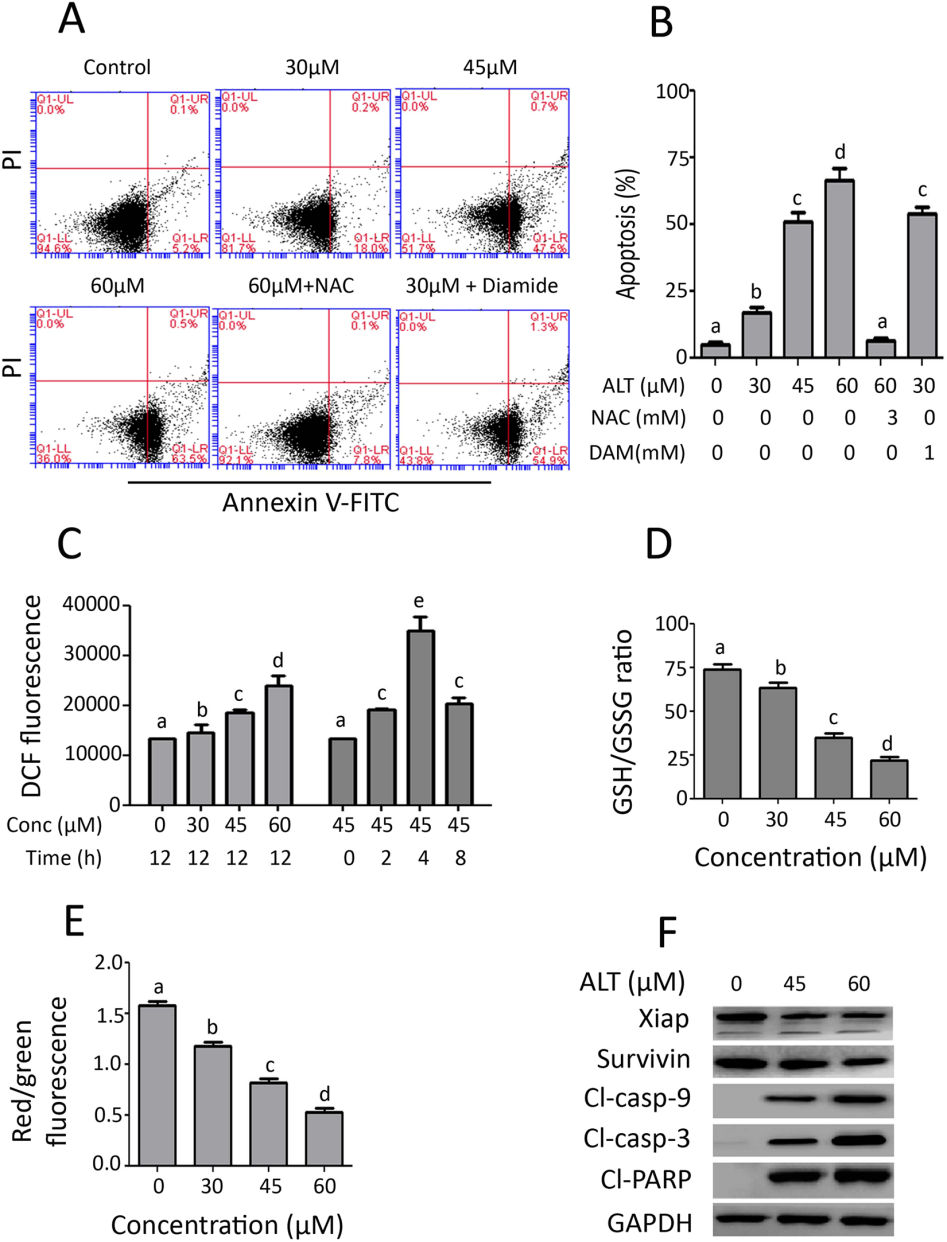
Effect of ALT on apoptosis, ROS generation, GSH/GSSG ratio and MMP dissipation. (**A**) A549 cells were treated with indicated concentration of ALT in the presence or absence of 3 mM NAC and 1 mM diamide for 12 h. Cells were stained with Annexin V/PI and apoptosis was determined by flow cytometery. (**B**) Statistical analysis of data from A. Columns not sharing same superscript letters within the same group differ significantly (*P* < *0*.*05*). (**C**) A549 cells were incubated with ALT in a dose- and time-dependent manner as indicated and ROS generation was determined by staining the cells with DCFH-DA. (**D**) A549 cells were treated with ALT for 12 h and intracellular GSH/GSSG ratio was measured according to kit instructions. (**E**) A549 cells were treated with ALT for 12 h and MMP was measured using JC-1 kit according to manufacturer instructions. Columns not sharing same superscript letters (Fig. **C**,**D**, and **E**) differ significantly (*P* < *0*.*05*). (**F**) A549 cells were treated with indicated concentrations of ALT for 12 h and expressions of xiap, survivin, cleaved caspases-9, cleaved caspases-3 and cleaved PARP were measured by Western blot analysis.

Data from live/dead assay indicate that ALT induces cell death in A549 and NCI-H1650 cells. To study the nature of cell death, we performed flow cytometry analysis of apoptosis by staining the cells with Annexin V-FITC and PI. As shown in Fig. 3A and B, ALT induces dose-dependent apoptosis in A549 cells. The ALT-induced apoptosis was effectively inhibited by ROS scavenger NAC while diamide enhanced it. However, NAC and diamide alone did not affect the viability of cells at indicated concentrations (data not shown). The data demonstrated that ALT induces apoptosis in A549 cells by targeting ROS generation. Therefore, we determined the intracellular ROS generation. We found that ALT increased intracellular ROS level in a dose-dependent manner. Time-dependent measurement of ROS showed that level of ROS generation was maximum at 4 h in response to 45 μM ALT treatment (Fig. 3C). Depletion of intracellular GSH level is an early hallmark in ROS-mediated apoptosis in multiple cancer cells^25^. GSH/GSSG ratio is critical for cellular redox potential. A decrease in GSH/GSSG ratio is a sensitive indicator of oxidative stress. Therefore, we measured GSH/GSSG ratio. ALT decreased intracellular GSH/GSSG ratio in a dose-dependent manner (Fig. 3D). Increased level of ROS and decreased level of GSH/GSSG ratio results in oxidative stress which ultimately leads to mitochondrial dysfunction. In parallel with oxidative stress, ALT dissipated mitochondrial membrane potential dose-dependently (Fig. 3E). These results were further verified using NCI-H1650 cells. A similar trend in ROS generation, GSH depletion and mitochondrial membrane potential disruption has been found in NCI-H1650 cells in response to ALT treatment (Supplementary Figure 1B–D). Moreover, ALT increased the expression of cleaved caspases-3,-9 and PARP (apoptosis markers) and decreased the expressions of Xiap and survivin (inhibitors of apoptosis proteins) in a dose-dependent manner in A549 cells (Fig. 3F). Taken together, the data demonstrate clearly that ALT induces oxidative stress-mediated apoptosis in lung adenocarcinoma cells.

### ALT induces oxidative stress-dependent ER stress in A549 cells

**Figure 4:**
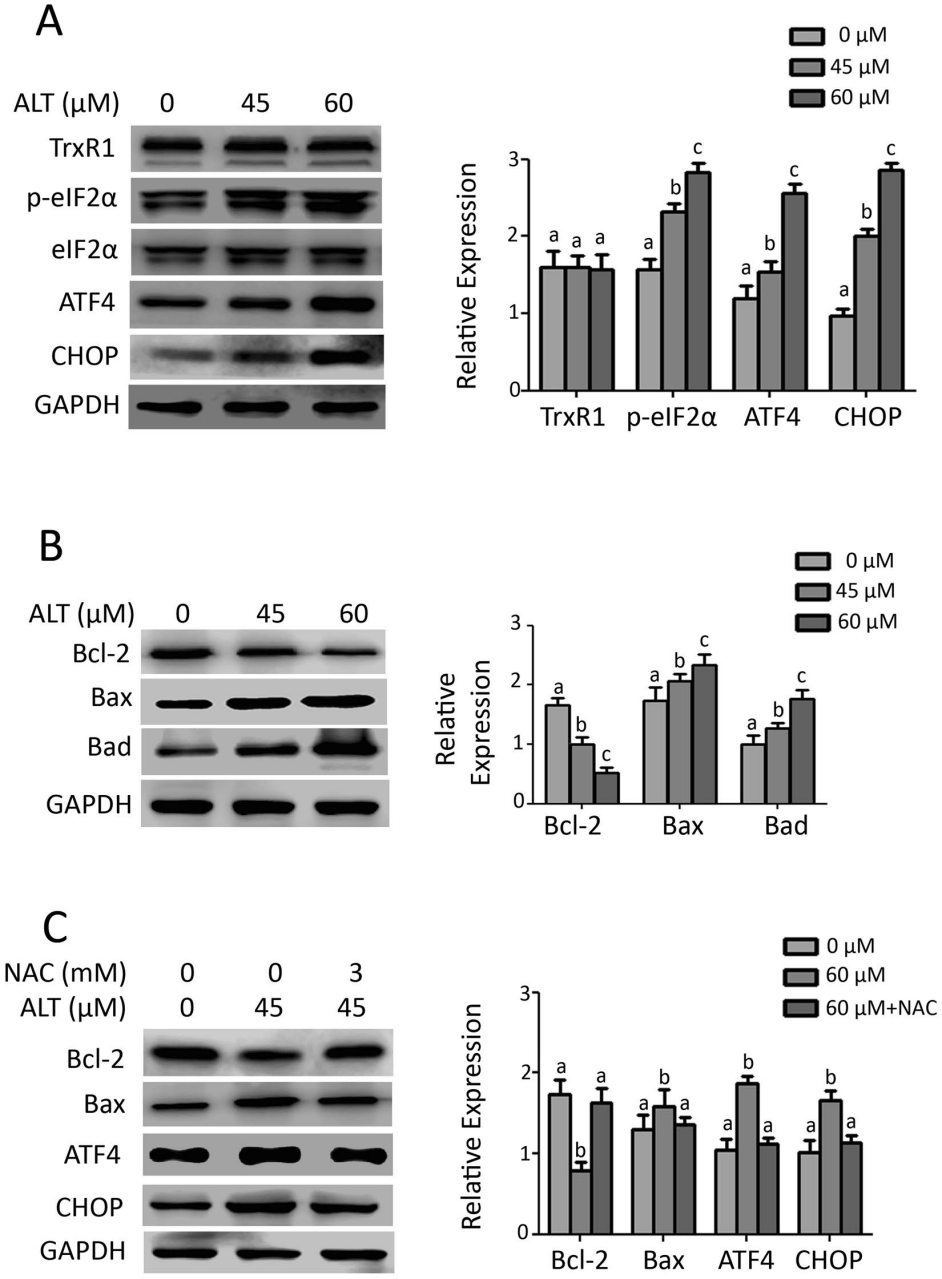
ALT induces ER stress and mitochondrial dysfunction through oxidative stress. (**A**) A549 cells were treated with indicated concentrations of ALT for 12 h. Total cell lysates were extracted and subjected to Western blots for the expression of TrxR1, p-eIF2α, eIF2α, ATF4 and CHOP. Columns not sharing same superscript letters within the group differ significantly (*P* < *0*.*05*). (**B**) A549 cells were treated with ALT as indicated for 12 h. Total cell lysates were extracted and subjected to Western blots for the expression of Bcl-2, Bax, and Bad. Columns not sharing same superscript letters within the group differ significantly (*P* < *0*.*05*). (**C**) A549 cells were treated with 45 μM ALT in the presence or absence of 3 mM NAC for 12 h. Total cell lysates were then subjected to immunoblotting for the expression of Bcl-2, Bax, ATF4 and CHOP. Columns not sharing same superscript letters within the group differ significantly (*P* < *0*.*05*).

Oxidative stress has been reported to induce endoplasmic reticulum (ER) stress response^17, 26^. Therefore, we measured the expression of ER stress related proteins by Western blot. The data demonstrated that ALT significantly (P < 0.05) induced the expression of p-eIF2α and ATF4 in a dose-dependent manner. Increased expression of ATF4 has been shown to induce the expression of pro-death transcriptional regulator, C/EBP homologous protein (CHOP), also known as growth arrest and DNA damage-inducible gene 153 (GADD153). Induction of CHOP expression has been considered as a hallmark in the commitment of ER-stress induced apoptosis^15, 27^. Therefore, we measured the expression of CHOP. As shown in Fig. 4A, ALT significantly (P < 0.05) enhanced the expression of CHOP in A549 cells dose-dependently. Pretreatment of cells with 3 mM NAC effectively suppressed ALT-induced expressions of ATF4 and CHOP (Fig. 4C) indicating that ALT exerts ER stress through ROS generation. Inhibition of thioredoxin reductase 1 (TrxR1) has been shown to induce ROS generation and ER stress^17^. Next we measured the expression of TrxR1 in response to ALT treatment. No change in the expression of TrxR1 was observed in ALT treated cells (Fig. 4A) indicating that ALT induces ROS generation and ER stress independent of TrxR1 activity.

### ALT modulates Bcl-2 family proteins expression in A549 cells through ROS generation

Disruption of MMP is considered as a characteristic feature of mitochondrial dysfunction^21, 25^. Above studies (Fig. 3E) has shown that ALT dissipates MMP in A549 cells. Therefore, we hypothesized that ALT could modulate the expression of Bcl-2 family proteins. The data demonstrated that ALT dose-dependently decreased the expression of anti-apoptotic Bcl-2 protein while increased the expressions of pro-apoptotic Bax and Bad proteins (Fig. 4B). Moreover, we found that ALT-mediated expressions of Bax and Bcl-2 were completely reversed by NAC (Fig. 4C), indicating that ALT modulates the expression of Bcl-2 family proteins through oxidative stress.

### ALT inhibits constitutive and inducible STAT3 activation in lung adenocarcinoma cells

**Figure 5:**
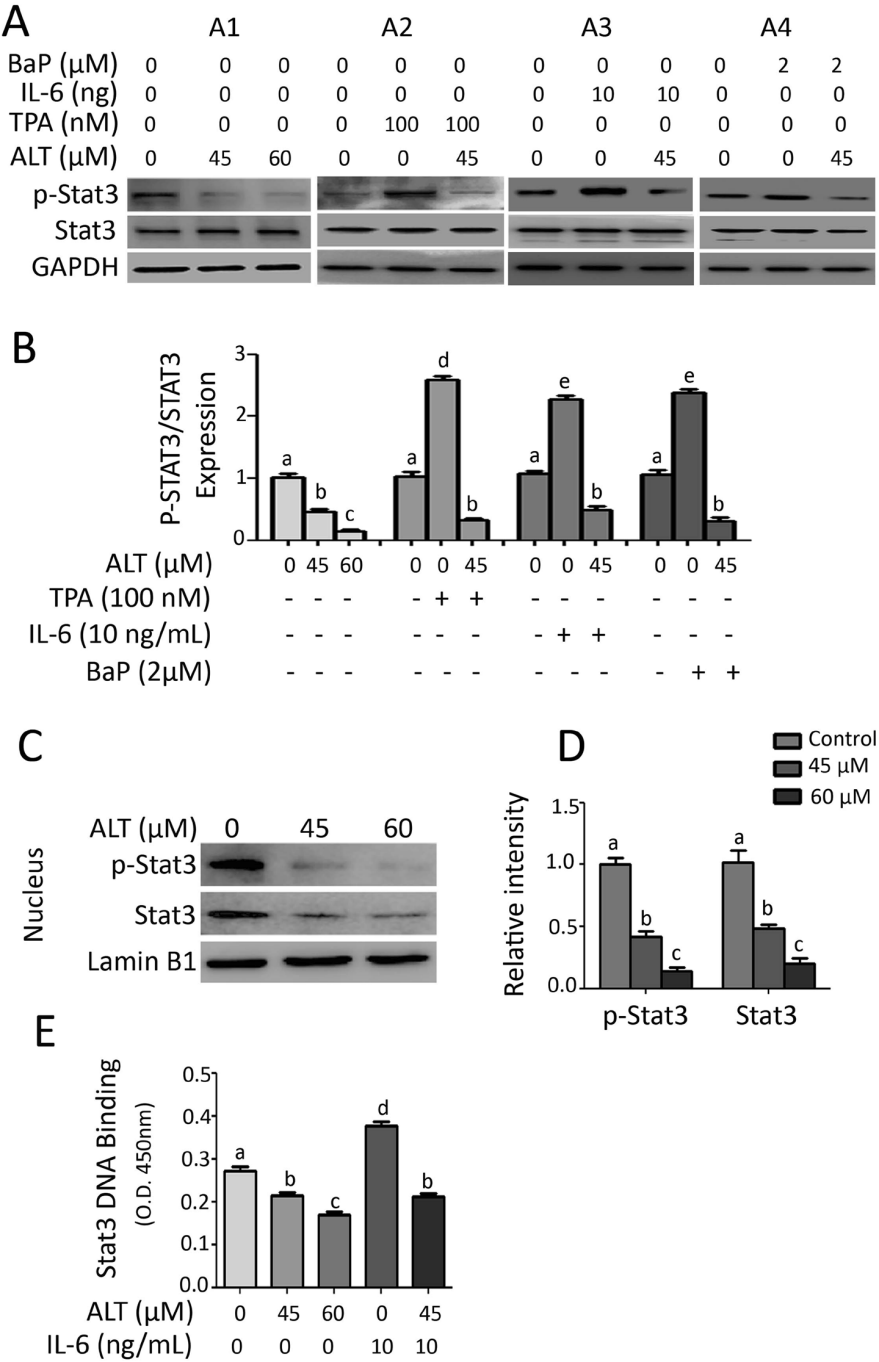
ALT inhibits STAT3 activation, translocation into nucleus and DNA binding activity in A549 cells. (**A**) ALT inhibits constitutive and inducible STAT3 activation (**A1**) A549 cells were treated with 45 and 60 μM ALT for 12 h, (**A2** & **A3**) A549 cells were pretreated with 45 μM ALT for 4 h and then stimulated with 100 nM TPA and 10 ng/mL IL-6 for 1 h respectively, (**A4**) A549 cells were treated with 2 μM BaP for 7 days and then treated with 45 μM ALT for 12 h. Total cell lysates were extracted and subjected to Western blot for the expression of p-STAT3 and STAT3. (**B**) Statistical analysis of p-STAT3/STAT3 expression. Columns not sharing same superscript letters within the group differ significantly (*P* < *0*.*05*). (**C**) A549 cells were treated with 45 and 60 μM ALT for 4 h. Nuclear proteins were isolated and subjected to Western blot for expression of p-STAT3 and STAT3. The expression of p-STAT3 and STAT3 was normalized with lamin B1. (**D**) Each bar in graph represents Mean ± SEM of three repeated experiments. Columns not sharing same superscript letters within the group differ significantly (*P* < *0*.*05*). (**E**) A549 cells were incubated with or without ALT for 4 h and then further incubated with IL-6 for 1 h. The nuclear extracts were then collected and assayed for STAT3 DNA binding activity according to the instructions of kit. Columns not sharing same superscript letters within the group differ significantly (*P* < *0*.*05*).

We have previously reported that ALT inhibits STAT3 activation in HepG2 cells^25^. Therefore, we evaluated the effect of ALT on STAT3 activation in A549 cells. STAT3 is activated by phosphorylation at Tyrosine 705 (Tyr705). Our data showed that treatment of cells with ALT for 12 h significantly (P < 0.05) suppressed the phosphorylation of STAT3 at Tyr705 in a dose-dependent manner. Next, we asked whether ALT could inhibit the inducible activation of STAT3. For this, we induced the activation of STAT3 using IL-6, TPA, and BaP. As shown in Fig. 5A, TPA (100 nM) and IL-6 (10 ng/mL) treatment for 1 h significantly induced STAT3 activation while pretreatment of cells with 45 μM ALT for 4 h effectively inhibited TPA- and IL-6-induced activation of STAT3 in A549 cells. Next we exposed the cells to 2 μM BaP (a major component of cigarette smoke) for 7 days and then treated with 45 μM ALT for 12 h. The data showed that BaP increased the phosphorylation of STAT3 while ALT inhibited the effect of BaP on STAT3 phosphorylation in A549 cells (Fig. 5A). Finally, we asked if ALT could inhibit STAT3 activation in other lung adenocarcinoma cells. The data demonstrated that ALT inhibited constitutive as well as inducible STAT3 activation in NCI-H1650 lung adenocarcinoma cells in a similar fashion (Supplementary Figure 1E and F). Moreover, ALT treatment decrease the phosphorylation of STAT3 in stimulated cells to a level even below than un-stimulated cells.

### ALT inhibits STAT3 translocation into nucleus and its DNA binding activity

Once STAT3 is activated, it translocates into nucleus where it binds with DNA and regulates expressions of various genes^28^. Therefore, we determined STAT3 translocation into nucleus and its DNA binding activity. The data showed that consistent with the inhibition of STAT3 activation, ALT attenuated translocation of STAT3 and its DNA binding in a dose-dependent manner (Fig. 5C–E).

### ALT inhibits STAT3 activation through S-glutathionylation

**Figure 6:**
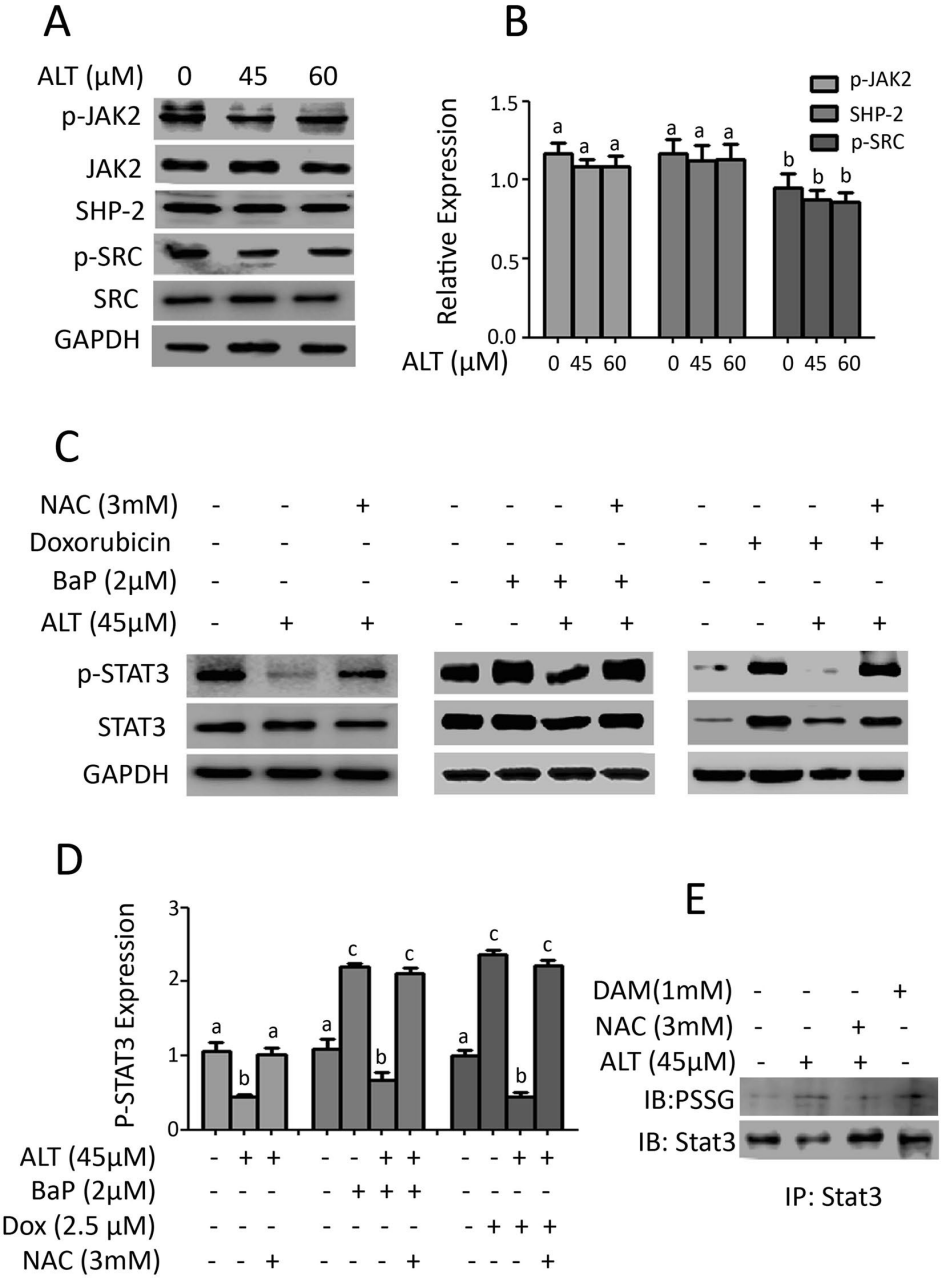
ALT abrogates STAT3 activation through oxidative stress. (**A**) A549 cells were incubated with ALT for 12 h. Cell lysates were collected and subjected to Western blot for the expression of p-JAK2, JAK2, SHP-2, p-SRC and SRC. (**B**) Protein expression was normalized to GAPDH. Each bar in graph represents Mean ± SEM of three repeated experiments. Columns not sharing same superscript letters within the group differ significantly (*P* < *0*.*05*). (**C**) A549 cells were incubated with or without BaP (2 μM) and Doxorubicin (2 μM) for 7 days. The cells were then treated with ALT in the presence or absence of NAC for another 12 h. Cell extracts were collected and expression of p-STAT3/STAT3 was evaluated by Western blot. (**D**) Each bar in graph represents Mean ± SEM of three repeated experiments. Columns not sharing same superscript letters within the group differ significantly (*P* < *0*.*05*). (**E**) A549 cells were treated with diamide and ALT in the presence or absence of NAC for 4 h. The total cell lysates were collected and subjected to immunoprecipitation (IP) for STAT3 and immunoblotting (IB) for protein linked GSH (PSSG) and STAT3. ALT increased the level of glutathionylated STAT3 as diamide while NAC inhibited ALT-induced STAT3 glutathionylation.

STAT3 is activated by various upstream signaling molecules including Janus activated Kinases (JAKs), Src family kinases and protein tyrosine phosphatases (PTPs)^13, 14^. To probe into in-depth molecular mechanism of ALT-mediated suppression of STAT3 activation, we determined the effect of ALT on activation of STAT3 upstream kinases in A549 cells. As shown in Fig. 6A and B, ALT did not decrease the phosphorylation of Jak-2 and Src significantly (P < 0.05). Since activation of STAT3 is negatively regulated by PTPs, we measured the expression of SHP-2. The data demonstrated that the expression of SHP-2 was not affected by ALT treatment in A549 cells (Fig. 6A and B). The set of data suggest that ALT inhibits STAT3 activation without affecting STAT3 upstream kinases and protein tyrosine phosphatases. Next, we wonder if ROS is involved in ALT-mediated inhibition of STAT3 activation. We measured the effect of ALT on inhibition of constitutive and inducible STAT3 activation in the presence of NAC. As shown in Fig. 6 C and D, pretreatment of cells with NAC not only abolished the inhibitory effect of ALT on constitutive STAT3 activation but also reversed ALT-mediated suppressive effect on inducible STAT3 activation in A549 cells. The data indicate clearly that ALT exerts suppressive effect on STAT3 activation through oxidative stress. Based on the above data, we hypothesized that ALT inhibits STAT3 activation via S-glutathionylation, a post-translational modification that results from oxidative stress due to decreased GSH/GSSG ratio. To test this hypothesis, we treated the cells with ALT and diamide (positive control) for 4 h in the presence or absence of 3 mM NAC and immunoprecipitated STAT3 protein was subjected to immunoblotting for protein linked GSH (PSSG) and STAT3. As shown in Fig. 6E, ALT increased the expression STAT3-linked GSH in a similar fashion as that of diamide. Interestingly, pretreatment of cells with NAC, reduced the level of STAT3-linked GSH in ALT-treated cells to a level of un-stimulated cells. The collective data from above experiments indicate clearly that ALT inhibits STAT3 activation through S-glutathionylation in A549 cells.

### ALT enhanced chemosensitivity of doxorubicin both in A549/DR and A549 cells

**Figure 7:**
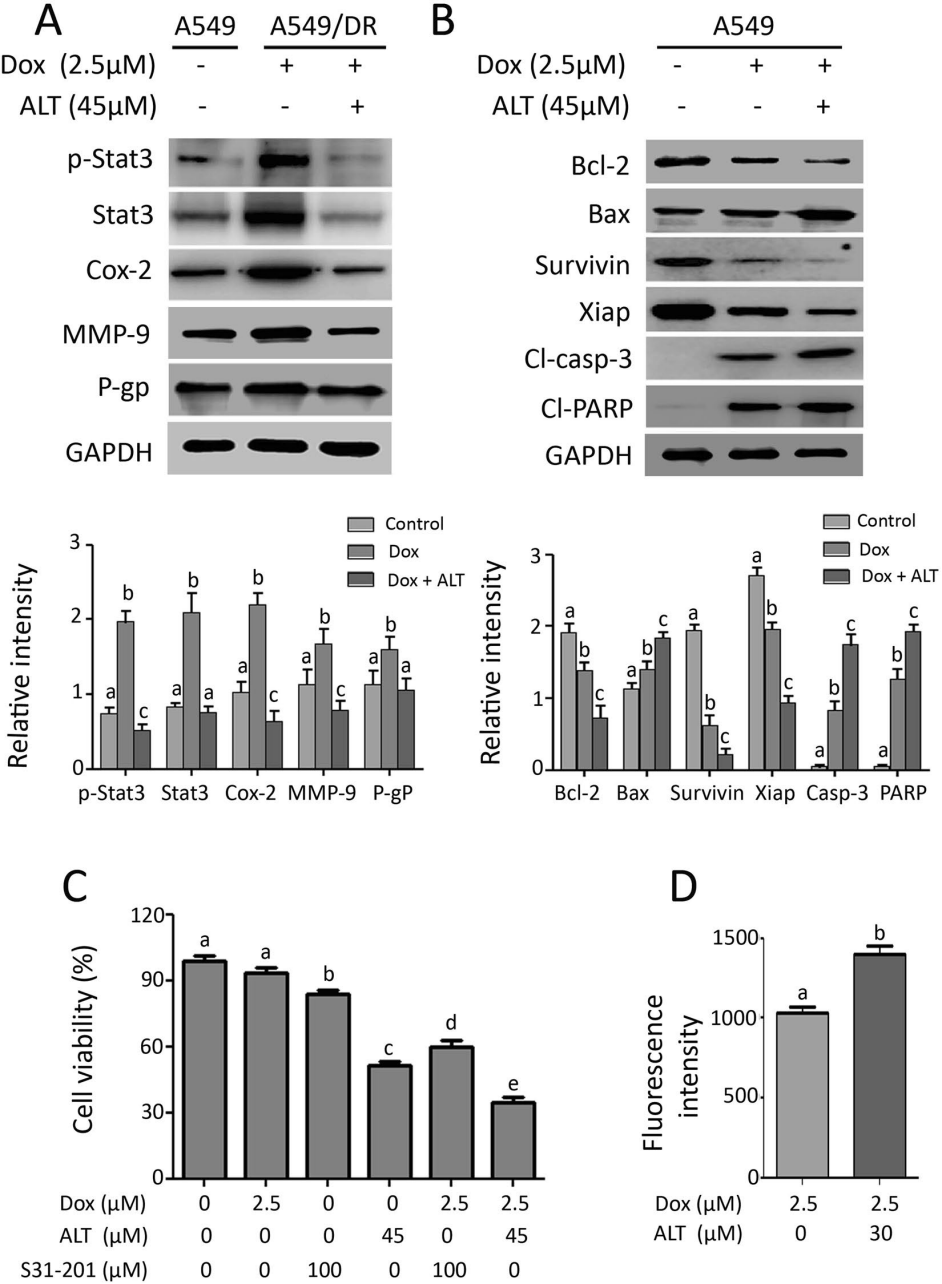
ALT augments doxorubicin toxicity in doxorubicin sensitive (A549) and doxorubicin resistant (A549/DR) lung cancer cells. (**A**) A549/DR cells were treated with doxorubicin or a combination of doxorubicin and ALT for 12 h. The expression of p-STAT3, STAT3, Cox-2, MMP-9 and p-gp was determined by Western blot. Columns not sharing same superscript letters within the group differ significantly (*P* < *0*.*05*). (**B**) A549 cells were treated with doxorubicin or a combination of doxorubicin and ALT for 12 h. The expression of Bcl-2, Bax, Survivin, Xiap, cleaved-caspase-3 and cleaved-PARP was determined by Western blot. Columns not sharing same superscript letters within the group differ significantly (*P* < *0*.*05*). (**C**) A549/DR cells were treated with doxorubicin, ALT and S31-201 either as single agent or in combination for 12 h and cell viability was determined by MTT assay. (**D**) A549/DR cells were incubated with or without 30 μM ALT for 12 h. The cells were then incubated with 2.5 μM doxorubicin for 2 h at 37 °C. After washing with PBS, the fluorescence of doxorubicin was measured at 488 nm by spectrophotometer. Columns not sharing same superscript letters differ significantly (*P* < *0*.*05*).

NSCLC cells including A549 cells are relatively insensitive to doxorubicin compared to SCLC cells. Moreover, doxorubicin has been shown to induce STAT3 activation and P-gp expression which are associated with doxorubicin-resistance in lung cancer cells^9, 29^. In the present study, we established doxorubicin-resistant “A549/DR” cell line as described in materials and methods section. After studying the efficacy of ALT as single agent in A549 cells, we sought to evaluate its effects in combination with doxorubicin in both doxorubicin resistant (A549/DR) and doxorubicin sensitive (A549) cells. Both A549/DR and A549 cells were treated with 45 μM ALT in combination with 2.5 μM doxorubicin, a concentration that was non-toxic to A549/DR cells while toxic to A549 cells. The IC_50_ value of doxorubicin against A549 cells was determined in our pilot experiment which was found to be 2.2 μM at 12 h drug treatment. As shown in Fig. 7A, doxorubicin remarkably enhanced the activation of STAT3 and expression of COX-2, MMP-9 and P-glycoprotein (P-gp) in A549/DR cells while ALT effectively inhibited the doxorubicin-mediated expressions of these proteins. On the other hand, in doxorubicin sensitive A549 cells, combine treatment of ALT with doxorubicin decreased the expressions of Bcl-2, Xiap, and survivin while increased the expressions of Bax, cleaved caspases-3 and cleaved PARP (Fig. 7B). Next, we were interested to know if inhibition of STAT3 activation and P-gp expression by ALT could reverse doxorubicin resistance in A549/DR cells. For this, we treated the A549/DR cells with 2.5 μM doxorubicin in combination with S31-201 (STAT3 inhibitor) or ALT for 12 h and viability of cells was determined by MTT assay. As shown in Fig. 7C, 2.5 μM doxorubicin did not affect viability of cells however, S31-201 (100 μM) and ALT (45 μM) reduced the viability of cells to 85% and 51% respectively. Moreover, combine treatment of doxorubicin (2.5 μM) with S31-201 (100 μM) and ALT (45 μM) significantly (P < 0.05) reduced the viability of cells to 60% and 34% respectively. The data suggest that suppression of STAT3 activation by S31-201 or ALT sensitizes cells to doxorubicin. As ALT inhibited the expression of p-gp in A549/DR cells, we measured the effect of ALT on intracellular accumulation of doxorubicin. Figure 7D shows clearly that ALT increased the intracellular accumulation of doxorubicin in A549/DR cells. The collective data indicate clearly that ALT overcomes doxorubicin resistance in A549/DR cells by suppression of STAT3 activation and inhibition of p-gp expression.

### ALT inhibits migration of A549 cells

**Figure 8:**
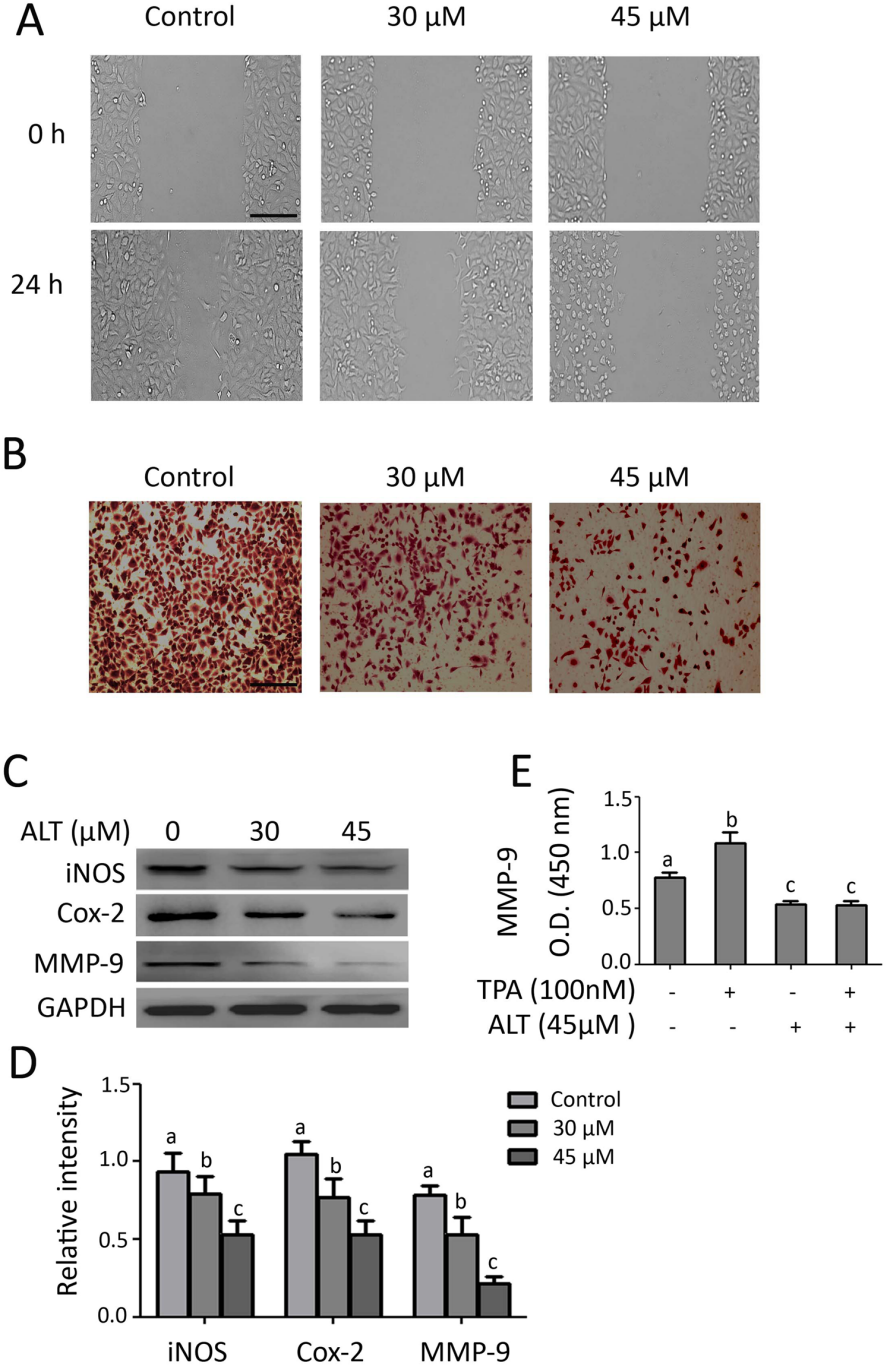
Effect of ALT on A549 cell migration. (**A**) A549 cells were cultured in 12 well plates. A wound was created in the middle using sterile micropipette tip. The cells were washed with PBS, exposed to ALT for 12 h. The drug containing medium was then replaced with fresh medium and allowed to grow at 37 °C for 24 h. The cells were photographed at 0 and 24 h under an inverted microscope. Scale bar = 100 μm (**B**). A549 cells were treated with ALT for 12 h. Drug containing medium was removed and cells were washed with PBS to remove floating cells. The adherent cells were harvested, counted and 2 × 10^4^ cells were seeded onto transwell chamber and incubated for 24 h as described in materials and methods. The migrated cells were fixed, stained with crystal violet and photographed. Scale bar = 100 μm (**C**) A549 cells were treated with ALT for 12 h and expression of iNOS, Cox-2, MMP-9 was measured by Western blot. (**D**) Statistical analysis of data from C. Columns not sharing same superscript letters differ significantly (*P* < *0*.*05*). (**E**) A549 cells were treated with ALT in the presence or absence of TPA for 12 h. The culture medium was collected and the level of MMP-9 in culture medium was measured according to kit instructions.

The effect of ALT on cell migration was evaluated by wound healing and Transwell chamber assays. The results of wound healing assay showed that cells migrated more quickly to heal the scratched wound in control group while ALT effectively suppressed the migration of cells toward scratched wound (Fig. 8A). The data of Transwell chamber assay further confirmed the anti-metastatic effect of ALT on A549 cells (Fig. 8B). In addition to evaluating the effect of ALT on migration ability, we also measured its effects on the expressions of inducible nitric oxide (iNOS), cyclooxygenase-2 (COX-2), and matrix metalloproteinase-9 (MMP-9), which are well known markers of cancer invasion and metastasis^23, 30, 31^. As shown in Fig. 8C and D, ALT dose-dependently reduced the expressions of iNOS, COX-2 and MMP-9 in A549 cells. In addition to measuring protein expression of MMP-9 in cells, we also measured the level of MMP-9 in culture media. TPA was used as a positive control. The data showed that ALT inhibited the secretion of MMP-9 into culture media (Fig. 8E).

## Discussion

Rational development of highly targeted drugs that kill cancer cells by interacting with a particular signaling molecule have been associated with sporadic responses due to activation of survival pathways and emergence of drug resistance^7, 13, 32^. Exploring new novel strategies for the effective treatment of cancer cells are therefore paramount. ROS-based drug development for effective treatment of cancer has emerged as a new paradigm in recent years^18, 33, 34^. We have previously showed that ALT, a sesquiterpene lactone compound induces ROS-mediated mitochondrial apoptosis in U87 glioblastoma and HepG2 liver cancer cells^21, 25^, however, the molecular targets and in-depth mechanism of ALT remains largely unexplored. In the present study, using A549 as a model cell line of NSCLC, we uncovered various novel molecular targets and cellular mechanisms which may help the further design and conduct of studies to develop ALT into ROS-based chemotherapeutic drug for effective management of NSCLC.

Firstly, we showed that ALT exerts an irreversible anti-proliferative effect in cancer cells and induces apoptosis via oxidative stress. Oxidative stress is caused by an increase in cellular ROS production and a decrease in cells antioxidant system’s activity. GSH and thioredoxin are two major antioxidant systems of the cells which play important role in detoxification of ROS and prevention and repair of ROS-induced damage to various cellular components including lipids, proteins and nucleic acids^35^. To get into further insight into ALT-induced oxidative stress, we measured the level of ROS and intracellular GSH/GSSG ratio and expression of thioredoxin reductase-1 (TrxR1), a major component of thioredoxin system. The data demonstrated that ALT increased ROS generation and decreased GSH/GSSG ratio without affecting the expression of TrxR1. Pretreatment of cells with NAC, (a precursor of GSH) completely reversed while diamide (GSH oxidizing agent) potentiated ALT-induced apoptosis. These findings suggested that ALT exerts oxidative stress by decreasing GSH level in A549 cells. The findings are in consistent with our previous reports^21, 25^ and that of Jiang *et al*.^36^, that ALT induces oxidative stress by depleting intracellular GSH.

Once oxidative stress is induced, it sets the cancer cells on the road to ruin by initiating diverse redox sensitive signaling cascades associated with apoptotic cell death. Mitochondria, the main component of intrinsic apoptotic machinery are the source as well as major target of ROS. Bcl-2 family proteins modulation, mitochondrial membrane potential disruption and cytochrome C release accompanied by caspases activation are characteristic features of mitochondrial-mediated apoptosis in cancer cells^37, 38^. In consistent with Bcl-2 family proteins modulation, ALT dissipated MMP, activated caspases-9 and -3 and increased the expression of cleaved PARP in A549 cells. In addition to mitochondrial dysfunction, increased level of ROS has been documented to induce ER stress-mediated apoptosis in various cancer types^39^. ER is an organelle primarily involved in calcium storage, folding, post-translational modification and export of newly formed proteins^39, 40^. Under normal circumstances, newly synthesized proteins are scheduled for inspection by a mechanism called “ER quality control” that ensures the fidelity of protein folding and post-translational modifications. However, ER homoeostasis is impaired by various detrimental stimuli such as glucose deprivation, calcium disruption and redox imbalance which results in an accumulation of unfolded or misfolded proteins in the ER, a process called as “ER stress”. ER stress is counteracted by unfolding protein response (UPR) which attempts to re-establish homeostasis and restore ER function by inhibiting general translation to reduce the accumulation of proteins in ER, by inducing the genes to increase the protein folding capacity and by activation of ER-associated degradation (ERAD) proteins to enhance clearance of unfolded proteins. While the UPR initially aims to restore ER functions and promotes cell survival, if ER stress persists or overwhelms, the UPR switches to initiate the process of cell death^41–43^. ER stress induces activation of several sensor proteins on ER membrane including PKR-like ER kinase (PERK). Once activated, PERK induces phosphorylation and inactivation of eIF2α which results in inhibition of protein synthesis. Thus phosphorylation of eIF2α acts as a key indicator of ER stress^28^. In the present study, ALT induced phosphorylation of eIF2α in A549 cells in a dose-dependent manner. While phosphorylation of eIF2α suppresses the translation of proteins, mRNA of ATF4 is preferentially translated into protein under such conditions. ATF4 induces the expression of CHOP, which is considered as a hallmark in the commitment of ER-stress induced apoptosis^41^. In line with established parameters of ER stress, ALT increased the expression of ATF4 and CHOP in A549 cells indicating the involvement of ER stress in A549 cells. Pretreatment of cells with NAC, completely reversed the ALT-induced expressions of Bax, Bcl-2, ATF4 and CHOP, validating the fact that ROS production is the major upstream regulator of ALT-induced mitochondrial dysfunction and ER stress in A549 cells. Consistent with the previous reports that ALT induces mitochondrial apoptosis in various cancer cells^21, 25, 36^, the induction of ER stress in response to ALT treatment has been reported for the first time in the present study.

Chemo-resistance appears to be a major challenge in the successful treatment of cancer^44^. The central role of STAT3 activation in the development of chemoresistance to classical drugs in cancer treatment is well established now^6, 45^. The identification of natural compounds able, on one hand, to suppress STAT3 activation and, on the other, to induce apoptosis in cancer cells may potentially be developed into therapeutic agents and use of such agents as a single agent or in combination with classical drugs suffering from drug resistance may potentially improve the cancer treatment outcomes. We and Chun *et al*., have recently reported that ALT inhibits STAT3 activation in HepG2 and MDA-MB-231 cells respectively^23, 25^. Using computational docking method, Chun *et al*., have shown that ALT selectively inhibits STAT3 activation probably by direct binding with SH2 domain of STAT3. STAT3 activation is regulated by various upstream signaling molecules including JAKs, Src and PTPs^13, 14^. In the present study, we found that ALT inhibits both constitutive as well as inducible STAT3 activation by multiple inducers including IL-6, TPA, BaP and doxorubicin in A549 cells. Consistent with inhibitory effect on STAT3 phosphorylation, ALT inhibited STAT3 translocation into nucleus and suppressed STAT3-DNA binding activity. However, ALT inhibited STAT3 activation without affecting STAT3 upstream kinases and protein tyrosine phosphatases. Several lines of evidence now suggest that STAT3 is a redox-sensitive transcriptional factor and its activation is also regulated at post-translational level through S-glutathionylation^46, 47^. S-glutathionylation process induces post-translational modification of cysteine residues on STAT3 protein which ultimately inhibits tyrosine phosphorylation. The exact molecular mechanism by which S-glutathionylation inhibits STAT3 phosphorylation remains elusive. It has recently been reported that S-glutathionylation of STAT3 could slightly modulate secondary and tertiary structure of STAT3. Therefore, it is speculated that conformational changes induced by binding of GSH with cysteine residues could in turn induce conformational change in phosphorylation site, thus hampering recognition of Tyr705 site by tyrosine Kinases^48^. As ALT-induced apoptosis and expressions of apoptosis regulators were fully reversed by NAC pretreatment, we hypothesized that ALT inhibits STAT3 activation through oxidative stress. To test our hypothesis, we measured the effect of ALT on STAT3 activation in the presence of NAC. In line with our hypothesis, pretreatment of cells with NAC not only abolished the inhibitory effect of ALT on constitutive STAT3 activation but also reversed ALT-mediated suppressive effect on inducible STAT3 activation in A549 cells. The findings provided clear evidence that ALT mediates suppressive effects on STAT3 activation through oxidative stress. To further confirm, we measured the expression of glutathionylated STAT3. ALT-induced expression of glutathionylated STAT3 was comparable to that induced by diamide and ALT-mediated STAT3 glutathionylation was reverted by NAC, providing clear evidence that ALT inhibits STST3 activation by oxidative stress-mediated S-glutathionylation process. To the best of our knowledge, this is the first report with evidence that ALT suppresses STAT3 activation through S-glutathionylation.

Suppression of STAT3 activation has been suggested to overcome drug resistance in various *in vitro* and *in vivo* studies^23, 46^. Herein our data provided evidence that STAT3 activation play vital role in development of doxorubicin resistance and ALT could potentially overcome doxorubicin resistance by inhibiting doxorubicin-induced STAT3 activation in A549/DR cells. STAT3 activation has been shown to induce drug resistance in various cancers through multiple mechanisms. Among various such mechanisms, induction of p-gp expression by STAT3 has been well documented^49, 50^. In line with published reports, we found higher expression of p-gp in A549/DR cells compared to A549 cells. Consistent with STAT3 inhibition, ALT decreased the expression of p-gp and increased the intracellular accumulation of doxorubicin in A549/DR cells. Taken together, the data demonstrated that ALT sensitizes A549/DR cells to doxorubicin by inhibiting STAT3 activation and p-gp expression. We further extended our study to evaluate the effect of ALT for possible combination therapy in A549 cells. We found that ALT in combination with doxorubicin decreased the expressions of Bcl-2, xiap and survivin and increased the expressions of Bax, cleaved caspases-3 and cleaved PARP. The data supports ALT as a potent candidate for drug development with advantages of being used in combination therapy to overcome drug resistance and to improve the efficacy of clinical drugs. To validate ALT as a potential therapeutic agent for the development of anticancer drug, we further evaluated its effect on cancer cell migration. ALT effectively inhibited the migration of A549 cells as evident from wound healing and Transwell chamber assays. Consistent with anti-metastatic effect, ALT suppressed the expressions of iNOS, COX-2 and MMP-9 which are well known markers of cancer metastasis. Our findings are in line with previous report demonstrating that ALT inhibits migration and suppresses expressions of COX-2 and MMP-9 in breast cancer cells^23^.

**Figure 9:**
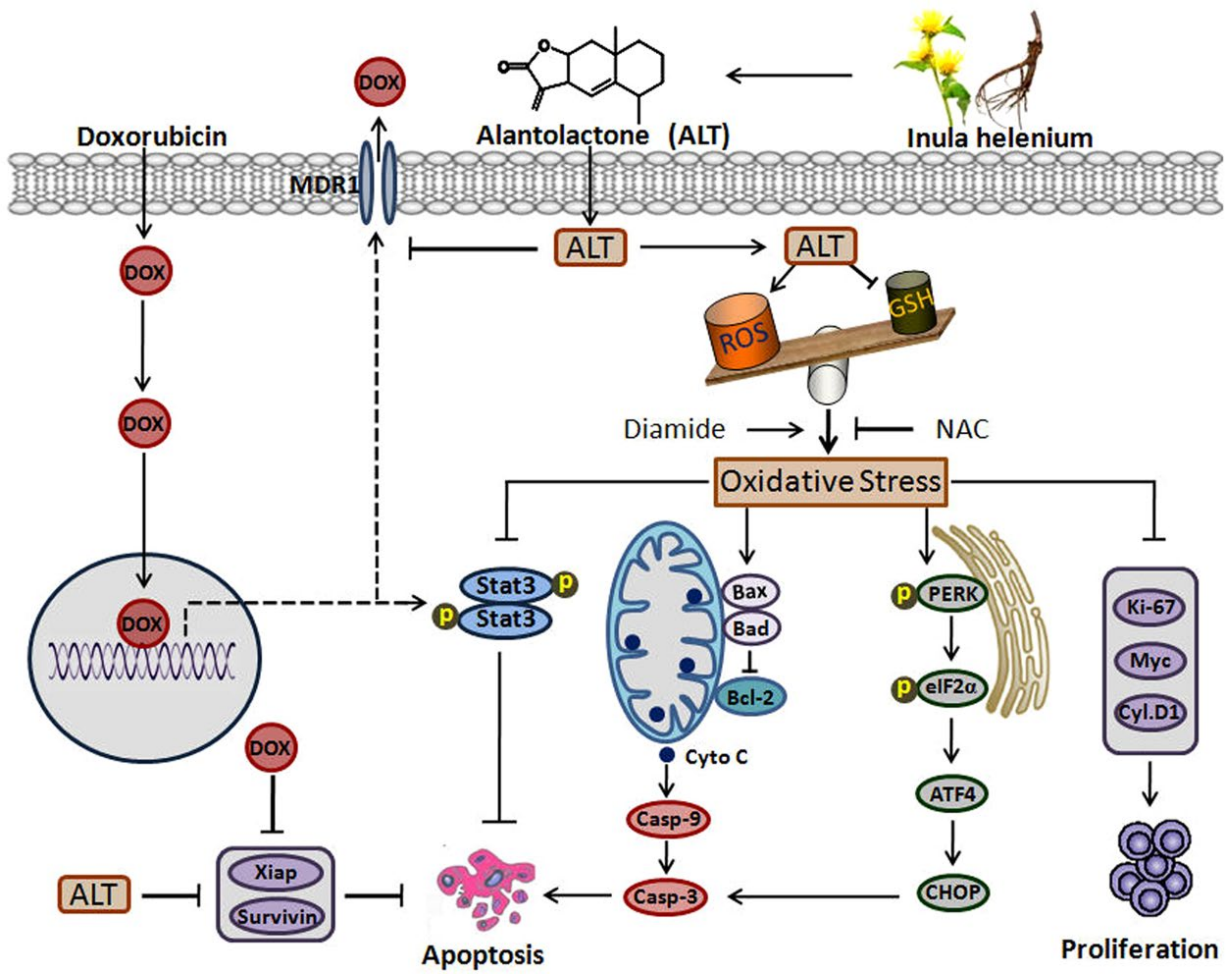
A schematic model for the molecular mechanism of ALT-induced anti-cancer activity in A549 lung adenocarcinoma cells.

In conclusion, we have demonstrated for the first time that ALT inhibits both constitutive and inducible activation of STAT3 by promoting STAT3 S-glutathionylation through oxidative stress. Induction of oxidative stress is the principal mechanism of ALT-mediated mitochondrial dysfunction, ER stress and apoptosis. Moreover, ALT enhanced chemosensitivity of A549 cells to doxorubicin and reversed doxorubicin resistance in A549/DR cells by inhibiting STAT3 activation and P-glycoprotein expression and increasing intracellular accumulation of doxorubicin. A schematic model for the molecular mechanism of ALT-induced anti-cancer activity in A549 lung adenocarcinoma cells has been shown in Fig. 9.

## Materials and Methods

### Materials

ALT (purity >98%) was purchased from Tauto Biotech (Shanghai, China). Dulbecco’s Modified Eagle’s Medium (DMEM) and fatal bovine serum (FBS) were obtained from Gibco (Eggenstein, Germany). Penicillin and Streptomycin were purchased from Solarbio co., Ltd. (Beijing, China). Annexin V-FITC apoptosis detection kit, ROS assay kit, mitochondrial membrane potential assay kit with JC-1, GSH/GSSG assay kit and Crystal violet staining solution were obtained from Beyotime Biotechnology (Nanjing, China). Diamide, 3-(4,5-Dimethylthiazol-2-yl)-2,5-Diphenyltetrazolium Bromide (MTT), N-acetyl-L-cysteine (NAC), propidium iodide (PI), calcein AM, Benzo(a)pyrene (BaP), dimethyl sulfoxide (DMSO), protease inhibitor cocktail, phenylmethylsulfonyl fluoride (PMSF) and Tris(2-carboxyethyl)phosphine hydrochloride (TCEP) were purchased from Sigma-Aldrich (St. Louis, MO). TPA (12-O-Tetradecanoylphorbol-13-Acetate) was purchased from Cell Signaling Technology while recombinant human interlukin-6 (IL-6) was purchased from PeproTech (Rocky Hill, USA). Doxorubicin and S31-201 were obtained from Selleckchem (Munich, Germany). Enzyme-linked Immunosorbent Assay (ELISA) kit for matrix metalloproteinase 9 (MMP-9) were purchased from Cloud-Clone Corp. (Houstan, USA) while TransAM^TM^ STAT3 Transcription Factor Assay Kit was purchased from Active Motif, Inc. (Carlsbad CA). The primary antibodies for cleaved caspases (3 & 9), cleaved PARP, p-STAT3 (Tyr705), STAT3, Cox-2, MMP-9, SHP-2, p-SRC, SRC were obtained from Cell Signaling Technology (Beverly, MA). The primary antibodies for Myc, Cyclin D1, Xiap, Survivin, CHOP, GAPDH, and Bad were purchased from Beyotime. The primary antibodies for Bax, Bcl-2, ATF4, eIF2α, TrxR1 and iNOS were obtained from Proteintech (Wuhan, China). The primary antibodies for JAK2, p-JAK2 and P-glycoprotein were obtained from abcam (Cambridge, MA) while p-eIF2α was obtained from Santa Cruz Biotechnology (Santa Cruz, CA) and glutathione monoclonal antibody from Virogen (Watertown MA). Horseradish peroxidase (HRP)-conjugated secondary antibodies (goat anti-rabbit, goat anti-mouse) were obtained from Sigma.

### Cell culture and treatment

Human A549 and NCI-H1650 lung adenocarcinoma cells were obtained from American Type Culture Collection (Manassas VA) and cultured in DMEM supplemented with 10% FBS, 100 units/mL penicillin and 100 μg/mL streptomycin at 37 °C with 5% CO_2_ in humidified atmosphere. Cells were treated with ALT dissolved in DMSO with final DMSO concentration of 0.5%. DMSO treated cells were used as control. Doxorubicin resistant A549 cells (A549/DR) were established by stepwise exposure to increasing concentration of doxorubicin from 100 nM upto 2.5 μM as described previously^9^.

### Determination of cell viability by MTT assay

A549 cells were treated with ALT as indicated and cell viability was determined by MTT assay as described previously^37^. Following treatment, cells were incubated with 10 μL MTT reagent (5 mg/mL) at 37 °C for 4 h. Subsequently medium was removed and 150 μL DMSO was added to dissolve farmazan crystals. The absorbance was measured at 570 nm (A_570_) by a microplate reader (Synergy neo HTS multimode microplate reader, BioTek) and percentage of cell viability was calculated as follows:

$${\rm{Cell}}\,{\rm{viability}}( \% )=({\rm{A}}570{\rm{sample}}-{\rm{A}}570{\rm{blank}})/({\rm{A}}570{\rm{control}}-{\rm{A}}570{\rm{blank}})\times 100$$

### Live/Dead assay

A549 and NCI-H1650 cells were treated with different concentrations of ALT in the presence or absence of NAC and TCEP for 12 h. Live/Dead assay was performed to quantify live and dead cells as described by us previously^21^. Briefly, cells were harvested, washed with phosphate-buffered saline (PBS) and incubated with 2 μM calcein AM and 4 μM PI for 20 min in the dark at room temperature. After washing, cells were resuspended in PBS and analyzed for the fluorescence of calcein and PI under fluorescence microscope (Leica, DMI 4000B). Finally 100 cells were counted microscopically from three different areas for the percentage of live and dead cells.

### Microscopic observation of cell morphology

A549 cells were seeded in 96 well plates and incubated for 24 h at 37 °C. The cells were treated with indicated concentrations of ALT for 12 h in the presence or absence of NAC or TCEP. Following treatment, cells morphological changes were observed and photographed by a phase contrast microscope (Leica, DMIL LED).

### Colony forming assay

A549 cells were treated with indicated concentrations of ALT for 12 h. The cells were washed, trypsinized and seeded into 6 well plates (500 cells/well) and allowed to grow into colonies for 7 days. The colonies were fixed with 4% paraformaldehyde (PFA) for 10 min and stained with crystal violet solution. The colonies were washed with PBS to remove extra stain and photographed. To quantify proliferation rate, methanol was added to each well to dissolve crystal violet stain and absorbance was measured at 595 nm.

### Apoptosis assay

A549 cells were treated with indicated concentrations of ALT in the presence or absence of 3 mM NAC or 1 mM diamide for 12 h. Following treatment, the cells were harvested, washed with PBS and resuspended in binding buffer containing 5 μL annexin V and 10 μL PI and incubated in the dark for 15 min according to kit instructions. The cells were analyzed by flow cytometry (BD Accuri C^6^) for the percentage of apoptotic cells.

### Determination of ROS generation

Cells were treated with ALT in a dose- and time-dependent manner and intracellular ROS generation was measured by ROS assay kit according to manufacturer’s instructions (Beyotime). Briefly, cells were harvested and incubated with 2′,7′-dichlorofluorescein-diacetate (DCFH-DA) for 30 min in the dark. After washing with DMEM for 3 times, DCF fluorescence was measured at an excitation wavelength of 488 nm and an emission wavelength of 525 nm by a fluorescence microplate reader (Synergy neo HTS multimode microplate reader, BioTek).

### Measurement of GSH/GSSG ratio

A549 and NCI-H1650 cells were treated with indicated concentrations of ALT for 12 h. The cells were collected and intracellular GSH/GSSG ratio was measured spectrophotometrically using GSH/GSSG assay kit (Beyotime) according to kit instructions.

### Determination of mitochondrial membrane potential (MMP)

MMP was determined by MMP assay kit (Beyotime). Briefly, A549 and NCI-H1650 cells were treated with ALT, washed with PBS and stained with JC-1 fluorescent probe according to manufacturer’s instruction for 20 min in the dark. After washing, the fluorescence distribution of JC-1 monomers (green fluorescence) and J-aggregates (red fluorescence) was measured by fluorescence microplate reader (Synergy neo HTS multimode microplate reader, BioTek). The MMP was calculated by a decrease in red/green fluorescence intensity ratio.

### STAT3 DNA binding assay

A549 cells were incubated with or without ALT for 4 h and then further incubated with IL-6 for 1 h. The nuclear extracts were collected and binding of STAT3 to DNA was measured with an ELISA-based TransAM^TM^ STAT3 assay kit according to manufacturer’s instructions.

### Measurement of MMP-9 in culture media

A549 cells were treated with ALT in the presence or absence of TPA for 12 h. The culture medium was collected and the level of matrix metalloproteinase-9 (MMP-9) in culture medium was measured with ELISA kit for MMP-9 (Cloud-Clone Corp) according to manufacturer’s instructions.

### Determination of intracellular doxorubicin accumulation

A549/DR cells were pretreated with 30 μM ALT for 12 h. Following treatment, the medium was removed and cells were washed with PBS. The cells were further incubated with 2.5 μM doxorubicin for 2 h at 37 °C. The cells were washed with PBS to remove extracellular doxorubicin, collected and the fluorescence of doxorubicin was measured at excitation/emission wavelength of 488/590 nm by fluorescence microplate reader (Synergy neo HTS multimode microplate reader, BioTek).

### Wound healing assay

A549 cells were grown to 80–90% confluency in 12 well plates. A wound was created by scratching the monolayer of cells with the help of a sterile 200 μL pipette tip. The cells were washed with DMEM to remove floating cells and exposed to ALT for 12 h in DMEM with 10% FBS. The drug containing medium was then replaced with fresh medium with 1% FBS and cells were allowed to grow for 24 h at 37 °C with 5% CO_2_ in humidified atmosphere. The cell migration was observed under microscope (Leica, DMI 4000B) and images were captured.

### Cell migration assay

Cell migration assay was conducted using Transwell chambers (8-μm pores; Corning Costar, Corning, NY, USA). A549 cells were treated with ALT for 12 h in DMEM with 10% FBS. Following treatment, cells were trypsinized and 2 × 10^4^ cells in 200 μL of medium with 1% FBS were seeded in the upper transwell insert chamber while 600 μL DMEM with 10% FBS was added into lower chamber as chemotactic factor. The cells were incubated for 24 h at 37 °C. After incubation, the non-penetrated cells from the upper side of transwell chambers were removed by cotton swabs. The penetrated cells were fixed with 4% PFA, stained with crystal violet solution, observed under microscope and photographed.

### Western blotting

After drug treatment, adherent and floating cells were collected, and whole cell lysates were prepared using cell lysis buffer containing 20 mM Tris (pH 7.5), 150 mM NaCl, 1% Triton X-100, 50 mM NaF, 0.1 mM PMSF, sodium pyrophosphate, β-glycerophosphate, EDTA, Na3VO4, and Leupeptin (Beyotime Biotechnology) on ice for 30 min. Nuclear proteins were extracted using ProteinExt^TM^ mammalian nuclear and cytoplasmic extraction kit (Transbionovo, Beijing, China). The protein concentration was determined by Enhanced BCA protein assay kit (beyotime). A total of 30 μg protein was separated on 8–12% sodium dodecylsulfate polyacrylamide gel electrophoresis (SDS-PAGE), and transferred to polyvinylidene difluoride (PVDF) membrane. After blocking with 5% skim milk, the membranes were incubated with respective primary antibodies overnight at 4 °C. After washing with Tris-buffered saline-Tween (TBST) solution, the membranes were incubated with HRP-conjugated goat anti-rabbit IgG or goat anti-mouse IgG secondary antibodies for 1 h at room temperature. After washing with TBST 3 times, the immunoreactive bands were detected by Immobilon Western chemiluminescent HRP substrate (Millipore, Billerica, MA) and chemiluminescence images were obtained using MicroChemi 4.2 imaging system (DNR Bio-Imaging system). Quantification of protein bands was performed using Image J software. GAPDH was used as loading control and detected in every individual blot. The bars in graph represent the relative density of the bands normalized to GAPDH from three repeated experiments.

### Immunoprecipitation and glutathionylation of STAT3

A549 cells were cultured in 10 cm^2^ cell culture plates and treated with 1 mM diamide and 45 μM ALT in the presence or absence of 3 mM NAC for 4 h. Cells were collected and lysed in IP lysis buffer (20 mM Tris (pH 7.5), 150 mM NaCl, 1% Triton X-100, 50 mM NaF, 0.1 mM PMSF, sodium pyrophosphate, β-glycerophosphate, EDTA, Na3VO4, and Leupeptin). 800 μg proteins from the clarified cell lysates were incubated overnight at 4 °C with rotation in the presence of STAT3 antibody (1:100 dilution). After incubation with sepharose A/G beads (Beyotime, Biotechnology) for another 1 h at 4 °C with rolling end-over-end, the immune complexes were collected, washed 3 times with cold lysis buffer and separated from the beads by boiling in LDS non-reducing sample buffer (ThermoFisher Scientific) for 5 min. The immunoprecipitated proteins were subjected to Western blotting for the detection of STAT3 and protein linked GSH (PSSG) using respective antibodies.

### Statistical analysis

The results are expressed as mean ± standard error mean (SEM) of 3 different experiments and statistically compared with control group or within the groups using one way ANOVA followed by Tukey’s Multiple Comparison Test. Student t-test was used to determine significance when only two groups were compared and P < 0.05 was considered statistically significant.

## Electronic supplementary material

Supplementary Information
