# Supplementary material for: Alantolactone induces apoptosis, promotes STAT3 glutathionylation and enhances chemosensitivity of A549 lung adenocarcinoma cells to doxorubicin via oxidative stress
Source: Sci Rep. 2017 Jul 24;7:6242. doi: 10.1038/s41598-017-06535-y (PMC5524969; doi:10.1038/s41598-017-06535-y)
Supplement: Supplementary file 1 — Supplementary Information [file 41598_2017_6535_MOESM1_ESM.pdf]

Alantolactone induces apoptosis, promotes STAT3 glutathionylation and enhances chemosensitivity of A549 lung adenocarcinoma cells to doxorubicin via oxidative stress.

Amara Maryam, Tahir Mehmood, He Zhang, Yongming Li, Muhammad Khan*, Tonghui Ma*

College of Basic Medical Sciences, Dalian Medical University, Dalian, Liaoning 116044, People's Republic of China.

Corresponding Authors: *Tonghui Ma, College of Basic Medical Sciences, Dalian Medical University, Dalian, Liaoning 116044, P.R. China. Tel:+86-411-86110278, Fax:+86-411-86110378, E-mail: tonghuima@dlmedu.edu.cn and Muhammad Khan, College of Basic Medical Sciences, Dalian Medical University, Dalian, Liaoning 116044, P.R. China. Tel: +86-411- 86118842; E-mail: khan_zoologist@ymail.com*

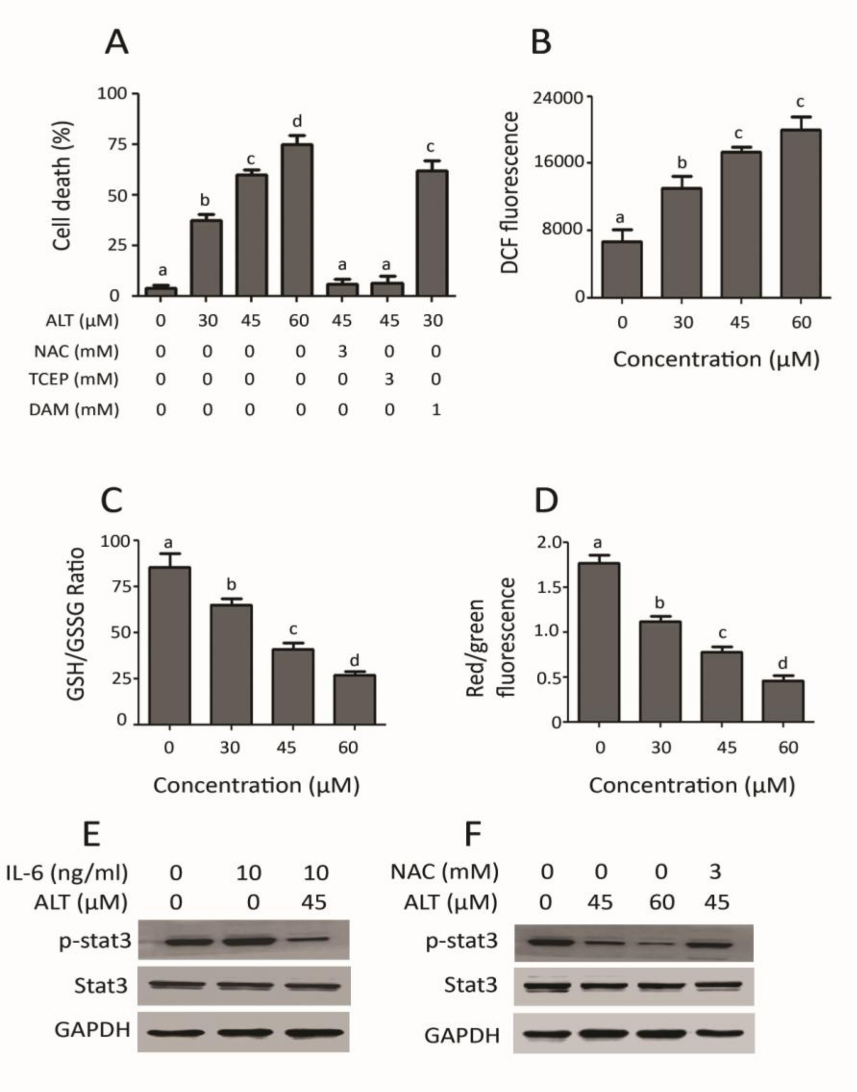

Supplementary Figure 1: ALT-mediated oxidative stress induces cell death and inhibits STAT3 activation in NCI-H1650 cells. **(A)** NCI-H1650 cells were incubated with indicated concentrations of ALT for 12 h and live and dead cells were quantified using Live/dead assay. Pretreatment of cells with 3 mM NAC and/or TCEP reversed while diamide (1 mM) potentiated ALT-induced cell death. (**B-D**) NCI-H1650 cells were treated with indicated concentrations of ALT for 12 h. Following treatment, cells were collected and ROS generation, GSH/GSSG ratio and mitochondrial membrane potential were measured using respective kits. Data from A-D are expressed as Mean±SEM (n=3). Columns not sharing same superscript letters differ significantly (*P<0.05*).

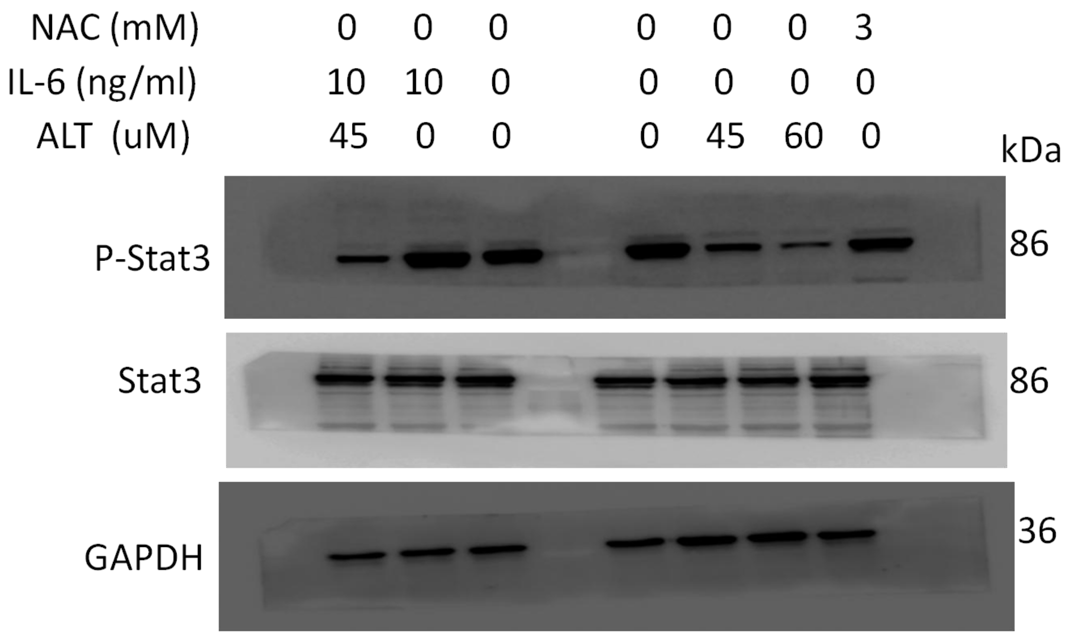

Supplementary Figure 1A: The original blots of supplementary figure 1E and F.

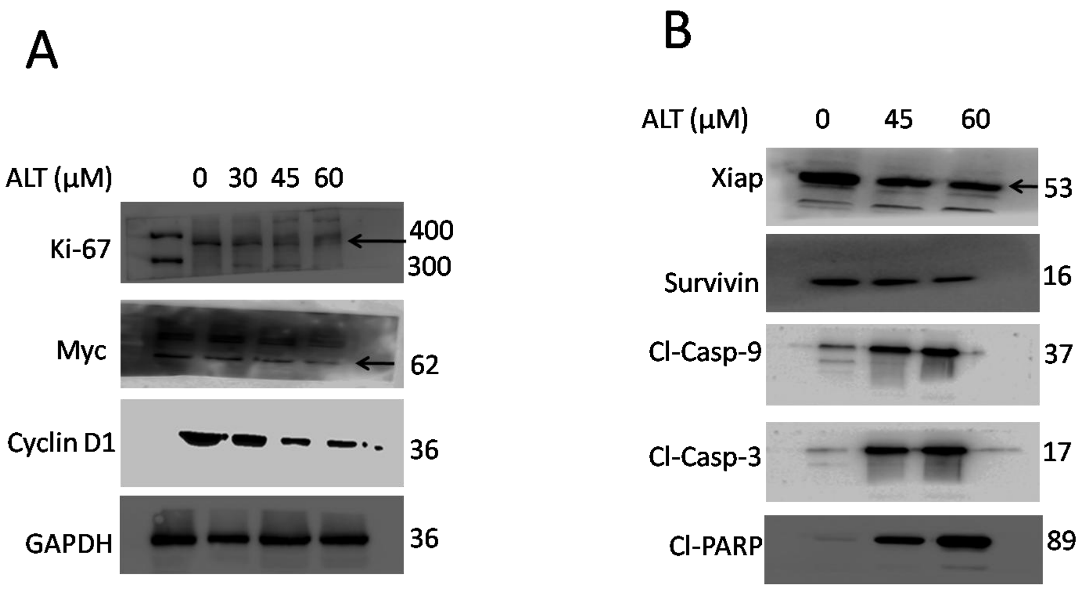

Supplementary Figure 2: The original blots of figure 2B **(A)** and 3F **(B).**

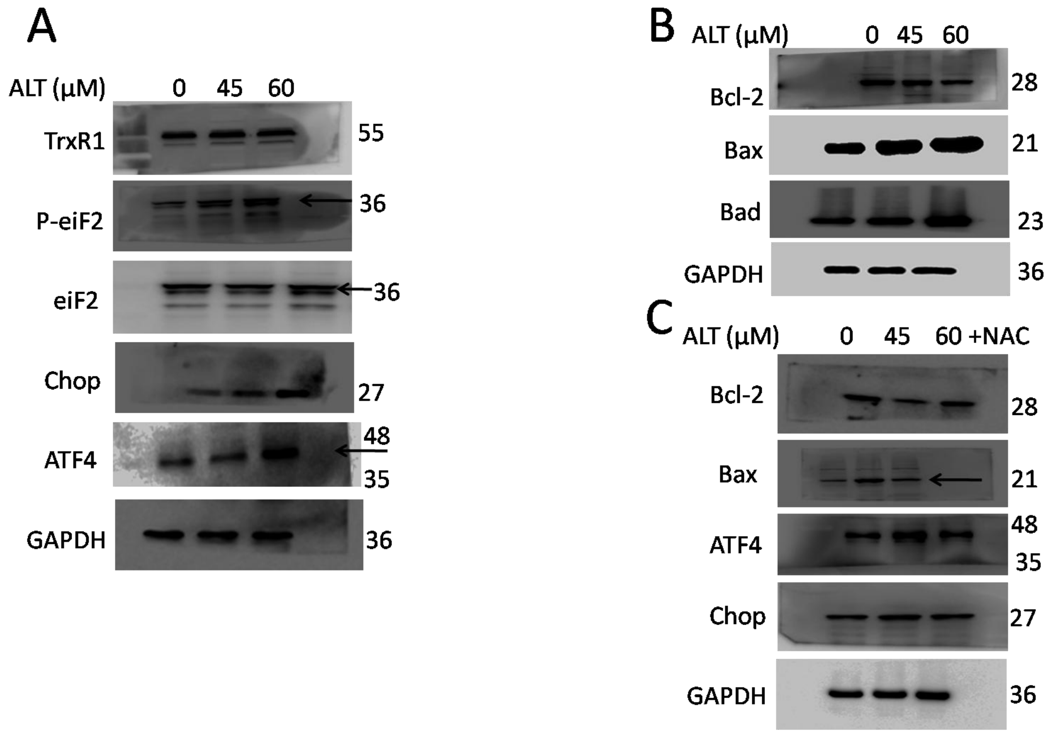

Supplementary Figure 2: The original blots of figure 4.

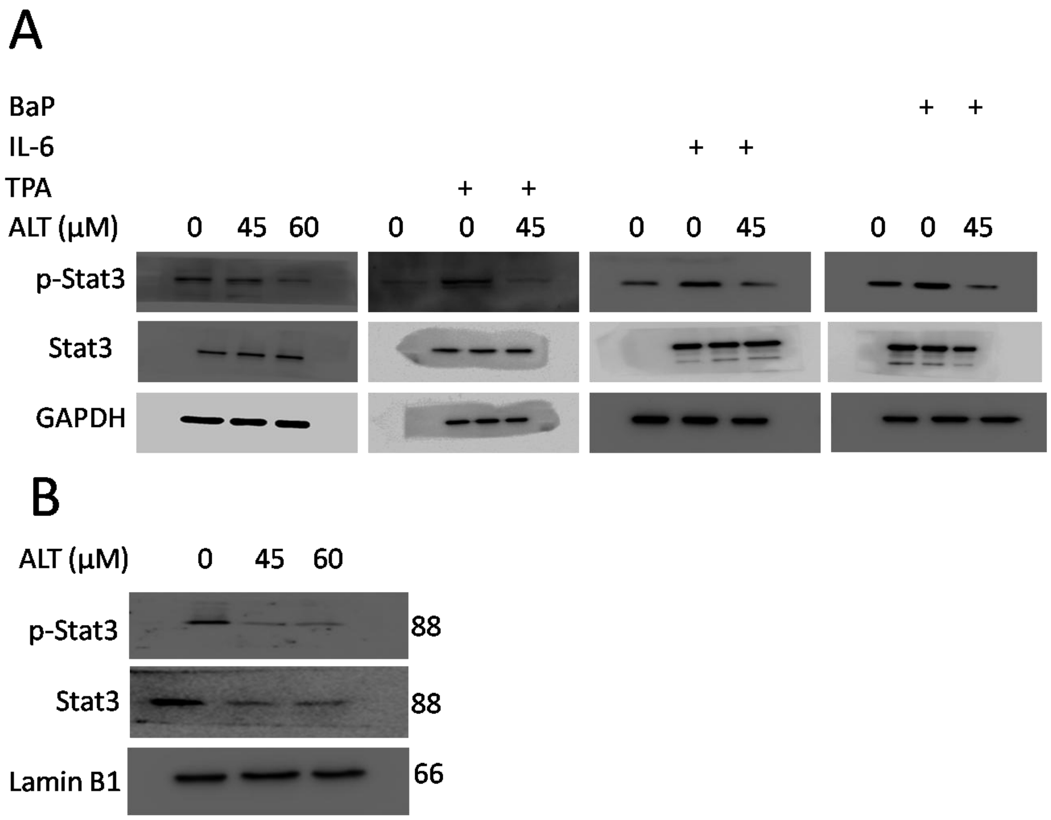

Supplementary Figure 3: The original blots of figure 5. **(A)** Original blots of figure 5A (A1-A4)**. (B)** Original blots of figure 5C.

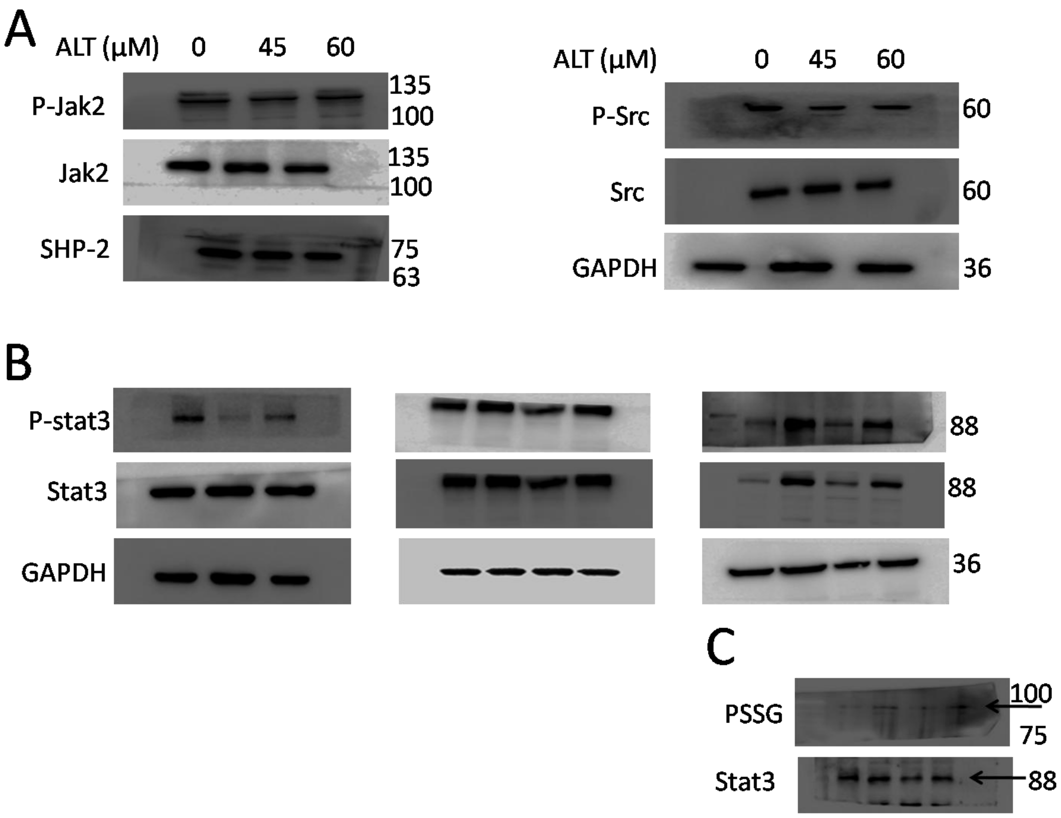

Supplementary Figure 4: The original blots of figure 6. **(A)** Represents figure 6A, **(B)** represents figure 6C, **(C)** represents figure 6E.

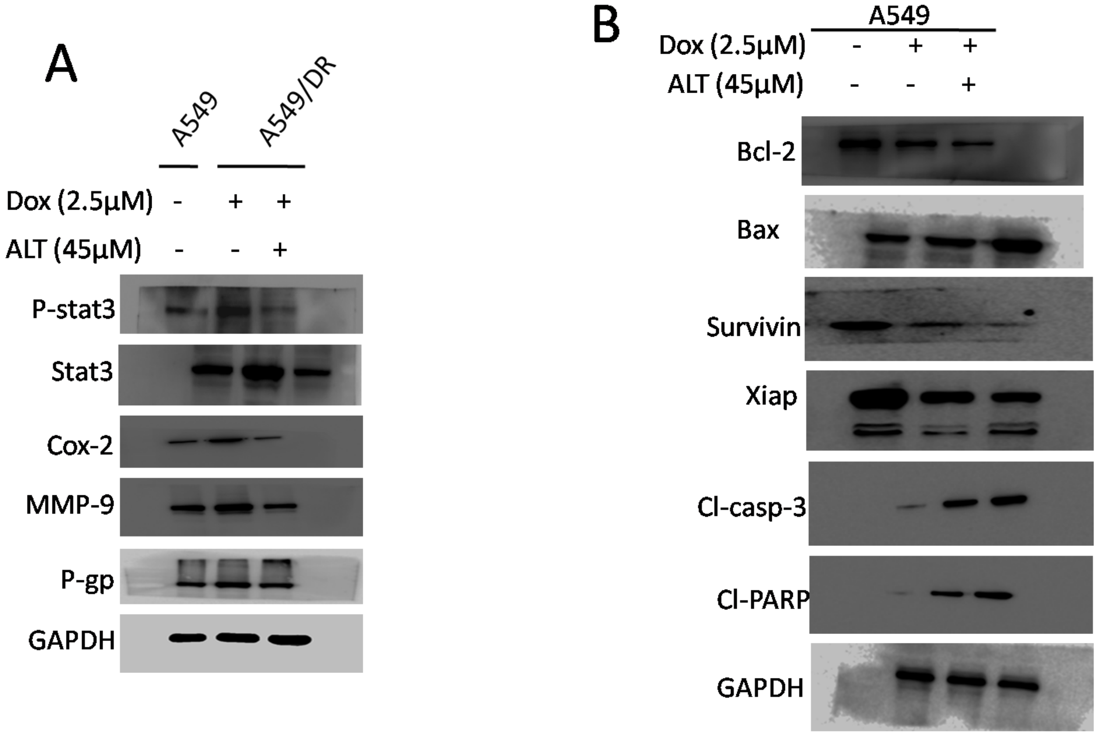

Supplementary Figure 5: The original blots of figure 7.

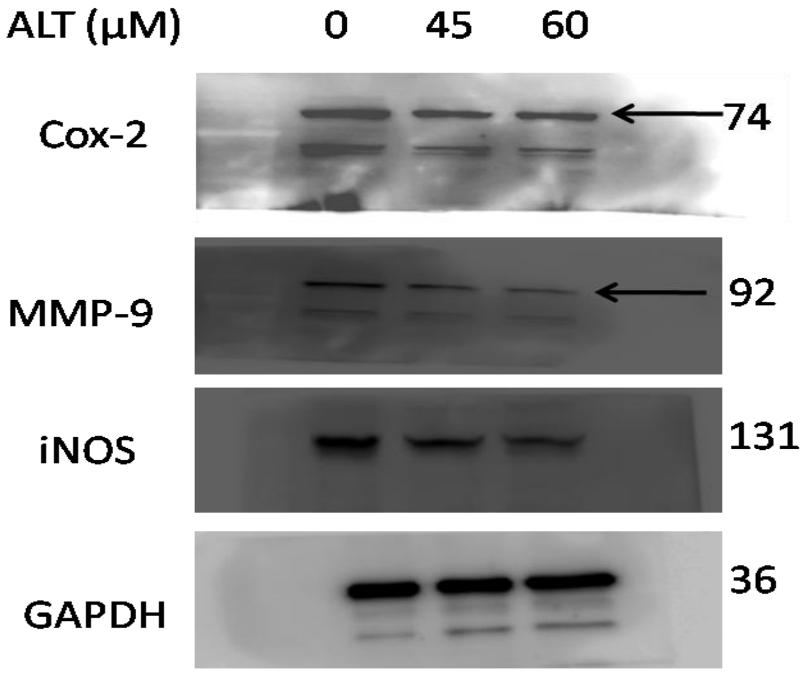

Supplementary Figure 6: The original blots of figure 8.
